# Aged hippocampal single‐cell atlas screening unveils disrupted neuroglial system in postoperative cognitive impairment

**DOI:** 10.1111/acel.14406

**Published:** 2024-11-14

**Authors:** Zizheng Suo, Ting Xiao, Yinyin Qu, Yuxiang Zheng, Wenjie Xu, Bowen Zhou, Jing Yang, Jie Yu, Hui Zheng, Cheng Ni

**Affiliations:** ^1^ Department of Anesthesiology, National Cancer Center/National Clinical Research Center for Cancer/Cancer Hospital Chinese Academy of Medical Sciences and Peking Union Medical College Beijing China; ^2^ State Key Laboratory of Molecular Oncology, Department of Etiology and Carcinogenesis, National Cancer Center/National Clinical Research Center for Cancer/Cancer Hospital Chinese Academy of Medical Sciences and Peking Union Medical College Beijing China; ^3^ Department of Anesthesiology Peking University Third Hospital Beijing China

**Keywords:** aged hippocampal cell atlas, dexmedetomidine, glia–neuron cycle, metabolic imbalance, neuroinflammation, postoperative cognitive impairment

## Abstract

Glia–neuron interaction is a crucial feature in aged hippocampus during the occurrence of postoperative cognitive impairment. However, the regulatory effects of microglia, astrocytes, and oligodendrocytes in this glia–neuron interaction, the potential mechanisms and gene targets are still to be elucidated. Here, single‐cell RNA sequencing was performed to detect the perioperative genomic expression characteristics of neuroglial system in the hippocampus of aged mice, and to investigate the potential cross‐cellular mechanisms and valuable treatment options for glia–neuron interaction‐related cognitive impairment. We found that postoperative neurons and glia cells exhibited protein dysmetabolism and mitochondrial electron misrouting. Impaired autophagy and circadian rhythm worsened microglia activation/neuroinflammation, and exacerbated these metabolic alterations. Reactive microglia also aggravated astrocyte and oligodendrocyte cytotoxicity through the PGD2/DP and complement pathways, altering glutamate level and synaptic function via the “tripartite synapses” model, and affecting neuronal myelination. Ligand‐receptor communication also indicated these synaptic and axonal dysfunctions via enhanced MDK and PTN pathways. Additionally, we found that anesthetic dexmedetomidine hold therapeutic potential within the disrupted neuroglial system. It enhanced neuronal metabolic rebalance (*Atf3*‐related) and reduced neuroinflammation from a multicellular perspective, therefore improving postoperative cognitive impairment. Together, our study proposes an aged hippocampal cell atlas and provides insights into the role of disrupted glia–neuron cycle in postoperative cognitive impairment. Our findings also elucidate the therapeutic potential and mechanism of dexmedetomidine intervention.

## Graphical abstract

In this study, we presented an aged hippocampal single‐cell atlas and explored the role of disrupted neuroglial system in postoperative cognitive impairment. Postoperative neurons and glia exhibited widespread protein dysmetabolism and mitochondrial dysfunction, which was exacerbated by microglia activation and neuroinflammation. Reactive microglia also aggravated astrocyte and oligodendrocyte cytotoxicity, altering glutamate level and synaptic function, and affecting neuronal myelination. Interestingly, perioperative DEX treatment can target neuronal metabolic disruptions, restoring the balance within neuroglial system and mitigating postoperative cognitive decline.

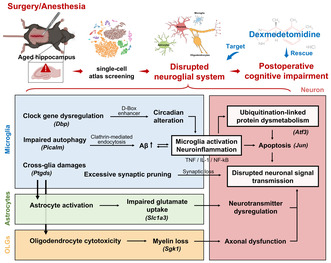

**Abbreviations**
AD: Alzheimer's disease
BP: Biological processes
DEGs: Differentially expressed genes
DEX: Dexmedetomidine
GO: Gene ontology
GSEA: Gene set enrichment analysis
PAPs: Perisynaptic astrocytes processes
POD 1: Postoperative day 1
RNA FISH: RNA fluorescent in situ hybridization
scRNA‐seq: Single‐cell RNA sequencing

## 1 INTRODUCTION

Postoperative cognitive impairment, recognized as a common complication of central nervous system in aged people, is characterized by impaired learning capacity, memory loss, and personality changes after surgery (Deiner et al., 2017). Hippocampal neuronal dysfunction is a crucial feature in this process, but little is known about its cross‐cellular mechanism and related treatment options. Our previous research indicated that metabolic disorder and blood–brain barrier damage are underlying pathogenic drivers (Suo et al., 2022). These processes involve complex regulation of neurons and various glia cells, including microglia, astrocytes, and oligodendrocytes (He et al., 2024). The neuroglial system refers to the collaborative network of these cells that maintain brain function and homeostasis, while the glia–neuron cycle describes their dynamic, reciprocal interactions. The disruption of the glia–neuron cycle observed in conditions like Alzheimer's disease (AD) and ischemia–reperfusion injuries, leads to a cascade of pathological changes (Lu et al., 2023; Sadick et al., 2022). Specifically, the fluctuation in microglial states, related to immune responses and lipid metabolism, can provoke Aβ‐associated neuronal death (Sun et al., 2023). Astrocyte morphological alterations contribute to synaptic dysfunction and impaired neurotoxin clearance (Endo et al., 2022). Whilst oligodendrocytes, sensitive to ischemic damage, exhibit halted maturation and delayed neuronal myelination (Ishibashi et al., 2012). These glia cells also interact with each other, and regulate APOE4 cascade in AD (Blumenfeld et al., 2024). However, there are few studies on this dysregulated glia–neuron cycle in the pathogenesis of postoperative cognitive impairment, which limits the understanding of perioperative hippocampal cytoarchitecture and heterogeneous glia–neuron regulatory properties.

This study undertook perioperative single‐cell sequencing in the hippocampus and attempted to uncover the role of integrated neuroglial system in postoperative cognitive impairment. Impaired autophagy and circadian rhythm promoted the transition of microglia from a homeostatic to a pathogenic state. Reactive microglia aggravated astrocyte and oligodendrocyte cytotoxicity, subsequently affected glutamate levels and synaptic function via “tripartite synapses” model, and influenced neuronal myelination. Therefore, the glia cells affected postoperative neuronal signal transmissions in the hippocampus. Dexmedetomidine (DEX)—based anesthesia regimen alleviated postoperative cognitive impairment (W. Wang, Ma, et al., 2022; Zeng et al., 2023), potentially by modulating neuroinflammation and immune cell activity (Gao et al., 2019; J. Wang, Xin, et al., 2022). However, additional research is required to understand the precise functional dynamics within glia–neuron cycle, to unravel the communications among these cells, their synergistic performance, and how DEX modulates these factors. In this study, we employed perioperative DEX intervention (simulation of clinical DEX combined anesthesia and analgesia protocol) and constructed a more comprehensive candidate therapeutic network targeting neuronal metabolic alteration.

## 2 MATERIALS AND METHODS

### 2.1 Animals

Animal experiments in this study were carried out in conformity with approved ethical protocols and guidelines from the Institutional Animal Care and Use Committee of Cancer Hospital Chinese Academy of Medical Sciences (No. NCC2021A040). Male C57BL/6 mice, 18‐month‐old, weighing between 23 and 34 g were used. The mice were housed in cages and maintained on a standard housing condition with food and water ad libitum for 2 weeks. Mice were randomly assigned to surgery group (surgery and anesthesia) and control group.

### 2.2 Surgery and anesthesia

Minimum alveolar concentration of sevoflurane for mice has been reported as 2.4%–2.7% (Li et al., 2014). In the present study, mice in surgery group received 2.5% sevoflurane in 50% oxygen for 30 min through breathing masks, and the control group received 50% oxygen for 30 min. The mice breathed spontaneously, and the sevoflurane concentration was monitored continuously with an anesthetic monitor (Datex, Tewksbury, MA, USA). Abdominal surgery was chosen for its association with significant cognitive impairment in mice, as well as aged patients. Our previous research showed that abdominal surgery minimally impacts motor performance, making it more suitable for cognitive assessments. The surgical procedure (exploratory laparotomy) was modified from previous studies and performed for the surgery group (Han et al., 2020). A longitudinal midline incision was made from xiphoid to 0.5 cm proximal pubic symphysis on the skin. The abdominal muscles and peritoneum, then approximately 10 cm of the intestine were exteriorized. The bowel loops remained outside the abdominal cavity for 1 min and then replaced into the abdominal cavity. The incision was finally sutured layer by layer with 5–0 Vicryl thread. Lidocaine gel was administered to provide postoperative analgesia. The entire procedure was completed under sevoflurane anesthesia. The rectal temperature was maintained at 37 ± 0.5°C, and this surgical protocol has been shown not to significantly alter values of blood pressure and blood gas in the preliminary studies. Then the mice were put into a chamber containing 50% oxygen until 10 min after the recovery of consciousness. After 24 h, mice were sacrificed and fresh hippocampal tissue was dissected for use (*n* = 3, sample size was similar to previous single‐cell studies that demonstrated sufficient statistical power [Arneson et al., 2018]). We selected postoperative day 1 (POD 1) as the sampling time point because it is both the period for memory formation during behavior test, and the point when major pathophysiological changes are most pronounced (Qian et al., 2023; Xu et al., 2022).

### 2.3 Tissue dissociation for single‐cell RNA sequencing (scRNA‐seq)

The Adult Brain Dissociation Kit (Miltenyi Biotec) was used to suspend hippocampus cells in 0.5% BSA‐PSA by digesting freshly dissected brain tissue with specific enzymes. To summarize, the rodent brain tissue was initially washed with cold D‐PBS then been cut into several slices in appropriate size with a scalpel. The samples obtained above were supposed to be blended with 1950 μL Enzyme mix 1 and 30 μL mix 2 successively in gentleMACS C Tube. Then, gentleMACS Program was performed. After termination of the program, detach C Tube from the gentleMACS Octo Dissociator with Heaters and centrifuge briefly to collect the sample at the bottom of the tube. The sample was resuspended and applied to a MACS SmartStrainer (70 μm) placed on a 50 mL tube. 10 mL of cold D‐PBS was added to the C Tube and applied onto the MACS SmartStrainer. After discarding MACS SmartStrainer and centrifuging cell suspension at 300 *g* for 10 min at 4°C, the supernatant was aspirated completely. Finally, Debris Removal Solution was applied and the final cell suspension solution was collected.

### 2.4 Single‐cell barcoding and library preparation

The scRNA‐seq libraries were prepared using manufacturer recommendations (Single Cell 3′ Reagent Kits v2 user guide; CG00052 Rev. F). On an 8‐well microfluidic chip, each cell was mixed with reagents and beads that carry a set of sequences including Illumina linker sequence, 16 bp 10X cell barcode, 10 bp UMI sequence (unique molecular identifier) and 30 bp poly dT, among which poly dT is considered a primer for reverse transcription of mRNA. The system constructed above was then wrapped by oil droplets to form GEMs (Gel bead in Emulsion). In each GEM, the mRNA was reverse transcribed into cDNA after cell rupture, and the cDNA from one GEM contained the same barcode which indicates from one cell. Subsequently, the cDNAs of all cells were collected and amplified and the library was constructed for the next step of sequencing.

### 2.5 scRNA‐seq and data preprocessing

The library molar concentration was quantified by Qubit Fluorometric Quantitation (ThermoFisher) and library fragment length was estimated by Bioanalyzer 2100 (Agilent). Sequencing libraries were loaded on an Illumina HiSeq2500 flow cell with sequencing settings following the recommendations of 10xGenomics. The Cell Ranger pipeline was designed to perform sample demultiplexing and to generate FASTQ files for read1, read2 and the i7 sample index. Read2, containing the cDNA, was mapped to the reference genome (mouse mm10) using STAR. Subsequent barcode processing, UMI filtering and single‐cell 3′ gene counting were performed using the Cell Ranger suite. Unsupervised clustering of the cells was performed using graph‐based clustering by means of principal component analysis. Clustering was visualized in two‐dimensional scatter plots (via tSNE).

### 2.6 Quality control of scRNA‐seq data

Quality control measures were implemented during sample preparation and sequencing to ensure data reliability. These included: (1) Cell viability in the suspensions exceeded 90%. (2) After library construction, initial quantification was performed using Qubit 2.0, and the insert size was confirmed with an Agilent 2100. The effective library concentration (2 nM) was verified by qPCR. (3) Sequencing data underwent stringent filtering to remove reads containing adapter sequences, reads with more than 10% unknown bases, and low‐quality reads (where bases with a quality score of Q ≤ 3 comprised over 50% of the read). Quality control results (Table S1–S2) showed zero undetermined bases, GC content around 46%, and 98% of bases with a quality score above 20, indicating a sequencing accuracy of 99%. (4) Alignment rates to the reference genome exceeded 95%. (5) Empty droplets, cell doublets, multiplets, and dying cells were excluded by filtering based on unique gene counts and mitochondrial gene percentages (Figure S2b–d).

### 2.7 Identification of cell clusters

To determine the cell types in each cluster, we examined the expression levels of cell type‐specific markers across each cluster and identified unique populations of neurons, microglia, astrocytes, oligoPCs, oligodendrocytes, endothelial, ependymal, T cells, neutrophils, and erythrocytes. Known markers for major hippocampal cell types were retrieved from Zeisel et al., 2015, Habib et al., 2016, and the Allen Brain Atlas (Lein et al., 2007).

### 2.8 Differentially expressed genes (DEGs) identification and functional enrichment analysis

**FIGURE 1:**
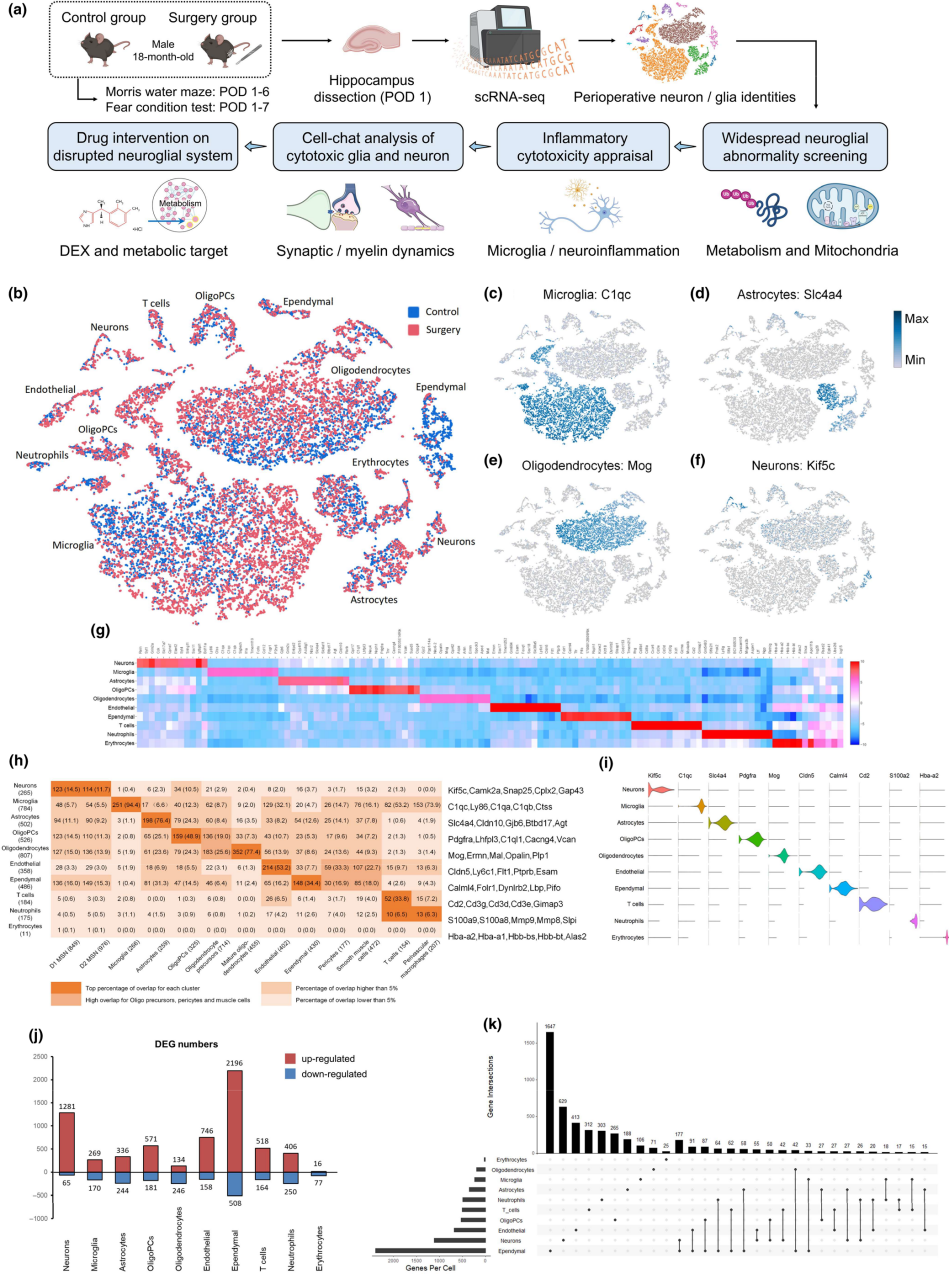
Aged hippocampal cell identities exhibit an activated gene expression pattern in the postoperative period. (a) The work flow of this study. (b) t‐SNE plot showing cell clusters. Each colored dot is a cell, with blue cells originating from control group and red cells originating from surgery group. (c–f) Cluster‐specific expression of known cell markers: C1qc for microglia, Slc4a4 for astrocytes, Mog for oligodendrocytes, and Kif5c for neurons. (g) Heatmap of top marker genes in 10 cell types. The deep red color means higher expression level. (h) Overlap between Drop‐seq defined marker genes of cell clusters (rows) with known cell type markers (columns) derived from previous studies. Marker gene numbers in each cell type are indicated in the parenthesis of the left and bottom. The numbers of overlapping genes are shown in the cells and statistical significance of overlap is indicated in the adjacent parenthesis. The deep color means higher percentage of overlap for each cluster. Top cell marker genes determined by our Drop‐seq data are listed on the right of the plot. (i) Normalized expression values of top cell marker genes are showed as violin plots with cell types as rows and genes as columns. (j) DEGs (*p* < 0.05 and |logFC| >0.1) between two groups within each cell cluster (up‐regulated in red and down‐regulated in blue). (k) The top 30 intersections of DEGs among 10 cell types shown in the upset plot. The left bar chart indicates total number of DEGs for each cell type, the bottom dark connected dots indicate substrates for each intersection, and the top bar chart indicates intersection sizes.

Within each identified cell type, surgery and control samples were compared for differential gene expression using Seurat's FindMarkers function. The genes had to be expressed in at least 1% of the single cells from one of the two groups. DEGs with an absolute logFC >0.1 were highlighted to emphasize the most significant differences. The overall distribution of DEGs was shown by bar plot and upset plot (Figure 1j,k). All DEGs with a Bonferroni‐corrected *p* value <0.05 were included to explore functional distinctions between oligodendrocyte progenitor cells and oligodendrocytes (Figure 4f). To predict putative biological functions, differential genes (*p* value <0.05) among two groups were used in the enrichment of Gene Ontology (GO) biological processes (BP) and Reactome pathways via g:Profiler (https://biit.cs.ut.ee/gprofiler/gost). Benjamini–Hochberg method was used to set the significance threshold (FDR <0.05). Enrichment networks were constructed using Cytoscape (v3.9.1) and the following plugins: Enrichmentmap (v3.3) constructed the correlation map of functional terms; AutoAnnotate (v1.3.5) automatically identified functional clusters and visually annotated them. The DEGs most relevant to the pathway functions and displaying the largest intergroup differences were selected as representative DEGs for the corresponding functional clusters. The top 5 DEGs (ranked by absolute value of logFC) in each major functional cluster were listed with their detailed data in Table S2. Drug targets of 3 anesthetics (DEX, sevoflurane, propofol) were added to the network using DrugBank database (https://go.drugbank.com/). Gene set enrichment analysis (GSEA) was also performed using GSEA software (v4.2.2). The number of permutations was set to 1000 and pathway database used during analysis was downloaded from https://baderlab.org/GeneSets.

### 2.9 Analysis of cell–cell communications

Perioperative cell–cell communications of hippocampus were analyzed by R package CellChat (v1.4.0). CellChat used gene expression data from cells as input and modeled the intercellular communication probability by integrating gene expression with prior knowledge of the interactions between signaling ligands and receptors (Jin et al., 2021). Specifically, communication probability between cell groups is modeled using the law of mass action, applied to ligand‐receptor interactions, incorporating gene expression profiles into a high‐confidence protein network from STRINGdb. This approach considers the multi‐subunit nature of ligands and receptors, costimulatory and co‐inhibitory effects. The communication probability here only represents the interaction strength and is not exactly a probability. The global and specific intercellular communication networks under physiological and postoperative conditions were quantified, visualized, and compared.

### 2.10 Drug treatment

The DEX intervention was achieved by administering a dose of 30 μg/kg DEX through intraperitoneal injection 30 min before surgery, 6 and 12 h after surgery (3 times in the perioperative period), enabling sustained intervention of DEX during perioperative period. The experimental mice were allocated into the following groups randomly (*n* = 6): Control group, intraperitoneally injected with 0.9% normal saline; Surgery group, underwent abdominal exploratory laparotomy and intraperitoneally injected with 0.9% normal saline; Surgery + DEX group, underwent abdominal exploratory laparotomy and intraperitoneally injected with DEX at the appointed time; DEX group, intraperitoneally injected with DEX and no surgery. The dosage of DEX in vivo was chosen based on the results of preliminary study.

### 2.11 RNA fluorescent in situ hybridization (RNA FISH)

Hippocampal tissues were collected 24 h postsurgery for the subsequent RNA FISH. Sliding microtome (Thermo Fisher Scientific, Tustin, CA, USA) was used to cut the paraffin‐embedded brains in coronal orientation. The brain sections (10 μm) underwent a thorough deparaffinization process with sequential immersion in xylene and ethanol. This was followed by permeabilization in 70% ethanol for over an hour. The sections were then washed in 1X PBS before a 20‐min incubation with proteinase K (10 μg/mL proteinase K in 1X PBS) at 37°C. Further washing steps were performed to prepare the tissue for hybridization. The hybridization step involved the preparation of a 125 nM probe solution, its application to the tissue section, and an incubation period of at least 4 h at 37°C. Subsequent steps included washing, DAPI nuclear counterstaining, and mounting with Vectashield Mounting Medium (50–100 μL). RNA in situ hybridization assay was performed according to manufacturer's instructions (Biosearch Technologies, Inc.). mRNA probes were designed and purchased from SAINTBIO (Beijing, China). Neurons, microglia, astrocytes, and oligodendrocytes were marked by Map2, Iba1, Gfap, and Mbp (5′‐Cy3), and the target genes were analyzed in these specific cell types with 5′‐FAM. Quantification of target genes was done using FIJI software to obtain the mean intensity and subtract background fluorescence (measured by obtaining the mean intensity of a small space immediately adjacent to the specific cell type being quantified).

### 2.12 Fear conditioning test and Morris water maze test

The Fear conditioning test (Xeye CPP, MacroAmbition S&T Development, Beijing, China) was used to assess the cognitive function of mice after surgery. Fear conditioning test consisted of a training process at postoperative day 1 (POD 1) and evaluations at POD 3 and POD 7. In the training process, mice were placed in the context chamber to acclimate for 180 s, then they received a pulsating tone (80 dB, 3600 Hz) for 60 s coterminated with a mild foot shock (0.8 mA, 0.5 s). In the evaluations, the hippocampus‐dependent memory was assessed by the freezing time during exposure to a novel context test (the test was performed in the same chamber but with no cues or shock), while the hippocampus‐independent memory was assessed by the freezing time during exposure to the tone stimulus (the test was performed in an alternative context and with no shock).

The Morris water maze test (Sunny Instruments Co. Ltd., Beijing, China) was used to assess the spatial learning and memory of mice after surgery. Morris water maze test consisted of a circular tank (120 cm in diameter and 50 cm high) containing water (23 ± 1°C) that is divided into four quadrants and a platform (10 cm in diameter) located 1 cm below the water in the target quadrant. In the place navigation test, the mice were placed in one quadrant facing the wall of the maze and allowed to explore the hidden platform for 90 s in each trial (four trials per day with an intertrial interval of 5 min). The time to locate the submerged plat form was recorded (defined as the escape latency). If the platform was not found within 90 s, the mice were guided to the platform, where they stayed for 15 s. Mice underwent daily testing in the water maze from day 1 to day 5 after surgery. In postoperative day 6, the submerged platform was removed from the water maze and a spatial probe test was performed for 90 s. The swimming speed, escape latency, times of platform crossing, and the time spent in target quadrant were recorded by a video camera.

### 2.13 Statistical analysis

Statistical analysis was performed with GraphPad Prism 7.0 software. Quantitative data are presented as the mean ± SD. Shapiro–Wilk test was used to assess normality of data, and test results exhibited a Normal/Gaussian distribution. Nonpaired two‐tailed Student's *t* test was used to identify significant differences between the two groups. One‐way ANOVA or two‐way ANOVA with Bonferroni's multiple comparison test were utilized to analyze significant differences between multiple groups. *p* < 0.05 was considered significant. The *p* value was adjusted with the FDR method (Benjamini Hochberg procedure).

## 3 RESULTS

### 3.1 Aged hippocampal cell identities exhibit an activated gene expression pattern in the postoperative period

The overall procedure is illustrated in Figure 1a. Sevoflurane anesthesia and exploratory laparotomy were performed in surgery group, and no perioperative stimulus in control group. Using Drop‐seq (Macosko et al., 2015), we finally sequenced 10,406 and 9959 hippocampal cells from surgery group and control group, respectively. They exhibited 13 distinct clusters in t‐SNE distribution (Figure 1b). Through cluster‐specific gene signatures, we eventually identified 8594 microglia (highlighted in Figure 1c with marker C1qc, spatial distribution in Figure S1–S5b), 2152 astrocytes (highlighted in Figure 1d with marker Sla4a4, positioned in hippocampal DG area as illustrated in Figure S1–S5c), 5861 oligodendrocytes (highlighted in Figure 1e with marker Mog, spatial distribution in Figure S1–S5e), 658 neurons (highlighted in Figure 1f with marker Kif5c, positioned in hippocampal CA area as illustrated in Figure S1–S5a), 1057 oligodendrocyte progenitor cells (oligoPCs, highlighted in Figure S3a with marker Pdgfra, spatial distribution in Figure S1–S5d), 460 endothelial cells (highlighted with marker Cldn5, spatial distribution in Figure S1–S5f), 1110 ependymal cells (highlighted with marker Calml4, spatial distribution in Figure S1–S5g), 271 T cells (highlighted with marker Cd2), 117 neutrophils (highlighted with marker S100a9), and 85 erythrocytes (highlighted with marker Hba‐a2). We summarized top cell marker genes (Figure 1g) and performed an overlap analysis (Figure 1h) with other brain scRNA‐seq libraries (Habib et al., 2016; Zeisel et al., 2015), confirming the most prominent marker genes in aged hippocampus (Figure 1i) and suggesting the reliability of our approach in distinguishing perioperative cell types. It should be noted that all the identified cell clusters were not due to technical or batch effects (Figure S2). None of the clusters were driven exclusively by a sample, as single cells from all samples are present in each cluster. Within/between‐group gene–gene correlations for each cluster also indicated overall coherence of gene expression between samples.

The aged hippocampal cells showed different t‐SNE distribution before and after perioperative stimulus, and the neuroglial types have high proportional abundance: microglia, 38.4% in control group and 45.8% in surgery group; astrocytes, 11.4% and 9.7%; oligodendrocytes, 28.4% and 29.2%; oligoPCs, 5.4% and 5%; neurons, 3.2% and 3.2% (Figure S3b,c). Oligodendrocytes were more vulnerable to excitotoxicity and interacted closely with neurons during surgical damages (Zhang et al., 2022), and we primarily focused on mature oligodendrocytes rather than oligoPCs in the following function analysis. This study focused on glia–neuron interactions in the main regions of hippocampus, and ependymal cells are located at the periphery of the hippocampus, lining the lateral ventricles. Neutrophils were relatively low in absolute number (with less than 100 cells per group), and this low count may contribute to data variability. Thus, this study did not focus on ependymal cells or neutrophils. Then, we identified DEGs between control and surgery groups to reveal surgery‐induced pathogenesis and related neuroglial types. The DEGs (|logFC| >0.1, *p* value <0.05) were mainly up‐regulated, which revealed an overall activated gene expression pattern in the postoperative period (Figure 1j). It may be related to the postoperative hippocampal changes in neuroinflammation, neuronal dysfunction and neurogenesis (Hovens et al., 2014). We showed the proportion of up‐regulated genes within neuroglial types. Neurons exhibited the highest proportion with 1281/65, ratio = 19.71. Microglia exhibited 269/170, ratio = 1.58. Astrocytes exhibited 336/244, ratio = 1.38. Only oligodendrocytes (134/246, ratio = 0.54) had mainly down‐regulated genes. Endothelial cells and oligodendrocytes exhibited the 2nd and 3rd highest proportion with 746/158, ratio = 4.72 and 2196/508, ratio = 4.32. The expression levels of top 5 DEGs were showed in the violin plot to indicate the surgery‐induced alterations (ranked by *p* value and logFC, Figure S3d). The upset plot showed top 30 intersections of DEGs among 10 cell types (Figure 1k). We found that the majority of DEGs were present in one specific cell type. (top 10 intersections), which suggested a relatively independent gene activation pattern in different hippocampal cell types.

### 3.2 Surgery triggers widespread neuroglial abnormalities in protein metabolism and mitochondrial function

**FIGURE 2:**
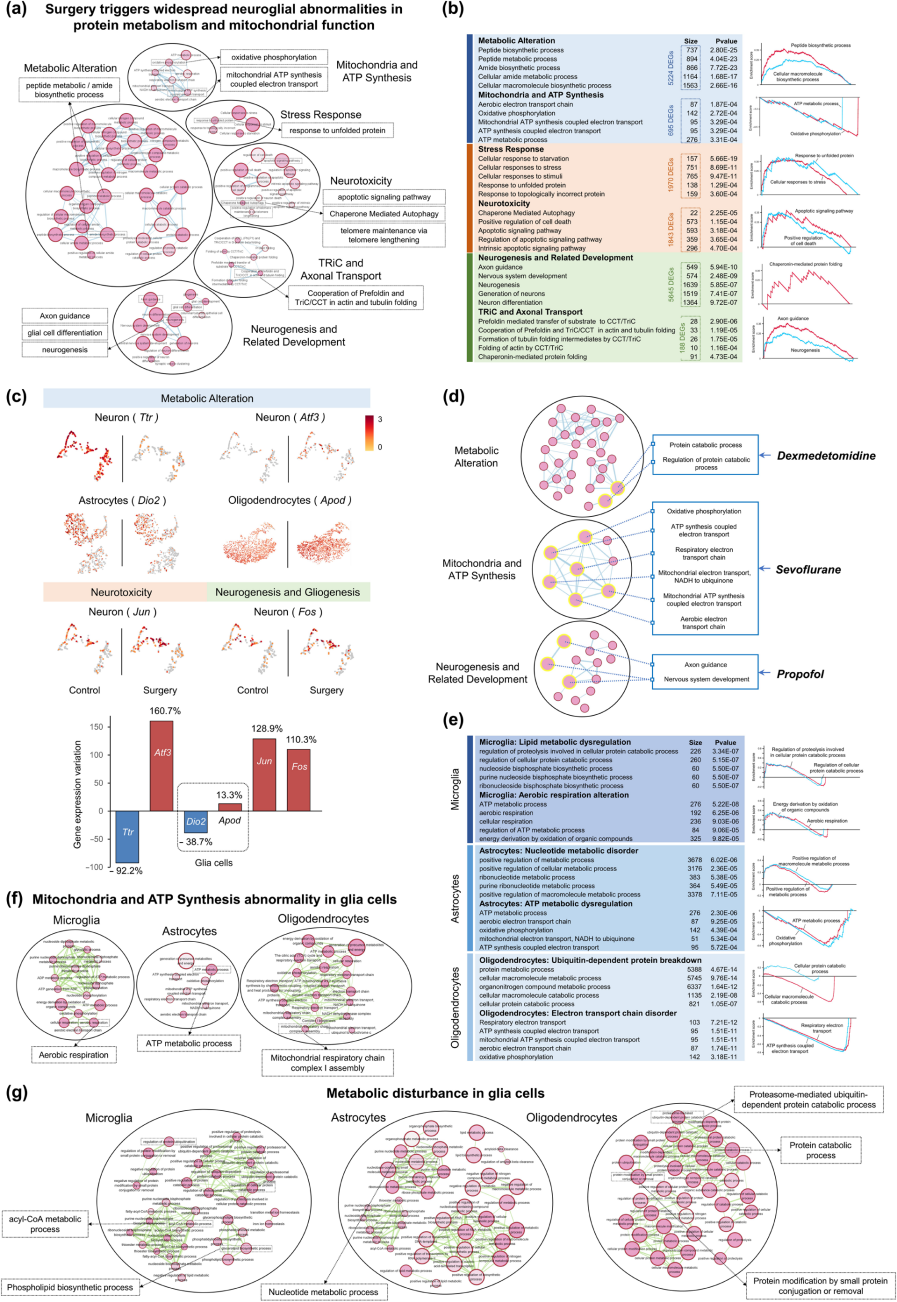
Surgery triggers widespread neuroglial abnormalities in protein metabolism and mitochondrial function. (a) Neurons‐related functional clusters. DEGs between control and surgery groups were used in the enrichment of Gene Ontology (GO) biological processes (BP) and Reactome pathways via g:Profiler (https://biit.cs.ut.ee/gprofiler/gost), and results were visualized by Cytoscape and its plugins (Enrichmentmap, AutoAnnotate). A circle (node) is a gene‐set (pathway) enriched in genes; bigger circle means larger size of gene set and deeper color means more significant. Edges (lines) represent genes in common between two pathways (nodes). A cluster of nodes represent overlapping and related pathways and may represent a common biological process. (b) The top 5 terms (ranked by *p* value) in each functional cluster showed in the chart with their DEGs counts (left). GSEA analysis of typical terms in each cluster (right). (c) The expression profiles of DEGs from functional clusters and changes in gene expression during perioperative period. (d) Pathological targets of three anesthetics (DEX, sevoflurane, propofol) using DrugBank database (https://go.drugbank.com/). (e–g) Glia cells also exhibited abnormalities in metabolism, mitochondria function and ATP synthesis.

To investigate what happened to neurons in the postoperative period, we used DEGs of neurons to construct a gene functional enrichment network. The BP terms and Reactome pathways in the network were grouped into six major functional clusters, and we listed the top 5 terms ranked by *p* value in each cluster and performed GSEA analysis to assess functional state of typical terms with the largest DEGs counts or the lowest *p* value (Figure 2a,b). The results indicated that Metabolic Alteration was the major cluster for the enriched terms in neurons (included 5224 DEGs, Figure 2b), including peptide metabolism and ceramide metabolism imbalance (shown in black dashed boxes of circle Metabolic Alteration, Figure 2a), which involved in multiple neurodegenerations. The DEG *Ttr* (thyroid hormone transport) and *Atf3* (ubiquitination‐linked protein dysmetabolism) were involved in this cluster (Figure 2c). The cluster Mitochondria and ATP Synthesis included oxidative phosphorylation, mitochondrial ATP synthesis coupled electron transport and related terms (695 DEGs). This energy deficiency could exacerbate stress response (1970 DEGs) and neurotoxicity (1843 DEGs), which included terms of ATP‐dependent protein misfolding, apoptosis, autophagy disorder, and telomere length instability. These neurotoxicity processes were related to ceramide and other metabolism imbalance. The DEG *Jun* (Figure 2c) was involved in cluster Stress Response and Neurotoxicity. Besides this, terms of neuron, glia, and endothelial cell differentiation and development were also extensively enriched, comprising the cluster Neurogenesis and Related Development (5645 DEGs). The DEG *Fos* (Figure 2c) was involved in this cluster, affecting actin filament/microtubule dynamics and axon guidance. Another cluster was comprised by TRiC complex‐related terms (188 DEGs), which also affected actin and tubulin folding. It may hinder axonal transport under mitochondrial dysfunction, and DEG *Atf3* was involved.

Then, we studied the possible neuronal intervention targets of three anesthetics (DEX, sevoflurane, and propofol) by using DrugBank database (https://go.drugbank.com/) and added the targets to functional enrichment network (Figure 2d). The results indicated that DEX was associated with the regulation of protein metabolism processes, which could be its primary protective mechanism in hippocampal neurons. Sevoflurane affected mitochondrial ATP metabolism and intracellular oxidative stress processes, while propofol affected neuronal axon growth, which could be their primary targets in neurons. The metabolic and mitochondrial changes in glia cells also may be involved in these neuronal damages (Figure 2e–g). Microglia showed lipid metabolic dysregulation including acyl‐CoA metabolic processes and phospholipid biosynthesis. The imbalance of these lipid metabolic products may affect neuronal protein metabolism by modulating protein kinases. Notably, the ATP synthesis was upregulated, contrasting with the energy deficit observed in neurons. Astrocytes showed nucleotide metabolic disorder, which may affect the secondary messenger cAMP and disrupt cell signaling (DEG *Dio2* was involved, Figure 2c). This nucleotide disorder may affect the recycling of neurotransmitters including glutamate and GABA. Besides, the ATP metabolic process and related terms were negative‐regulated, which may cause energy shortage just as neurons. Oligodendrocytes showed ubiquitin‐dependent protein breakdown, along with protein modifications through small molecule conjugation or removal (DEG *Apod* was involved, Figure 2c). Mitochondrial respiratory chain complex I assembly was enriched in oligodendrocytes, which indicated the decreased electron transport. For the above six characteristic DEGs (*Ttr*, *Atf3*, *Dio2*, *Apod*, *Jun*, and *Fos*) in neurons and glia cells, we showed their changes in the expression levels and distribution after surgery (Figure 2c, Figure S3e). *Ttr* and *Dio2* decreased by 92.2% and 38.7%, while *Atf3*, *Apod*, *Jun*, and *Fos* increased by 160.7%, 13.3%, 128.9%, and 110.3%, respectively.

### 3.3 Activated microglia aggravates hippocampal inflammatory cytotoxicity and metabolic imbalance

**FIGURE 3:**
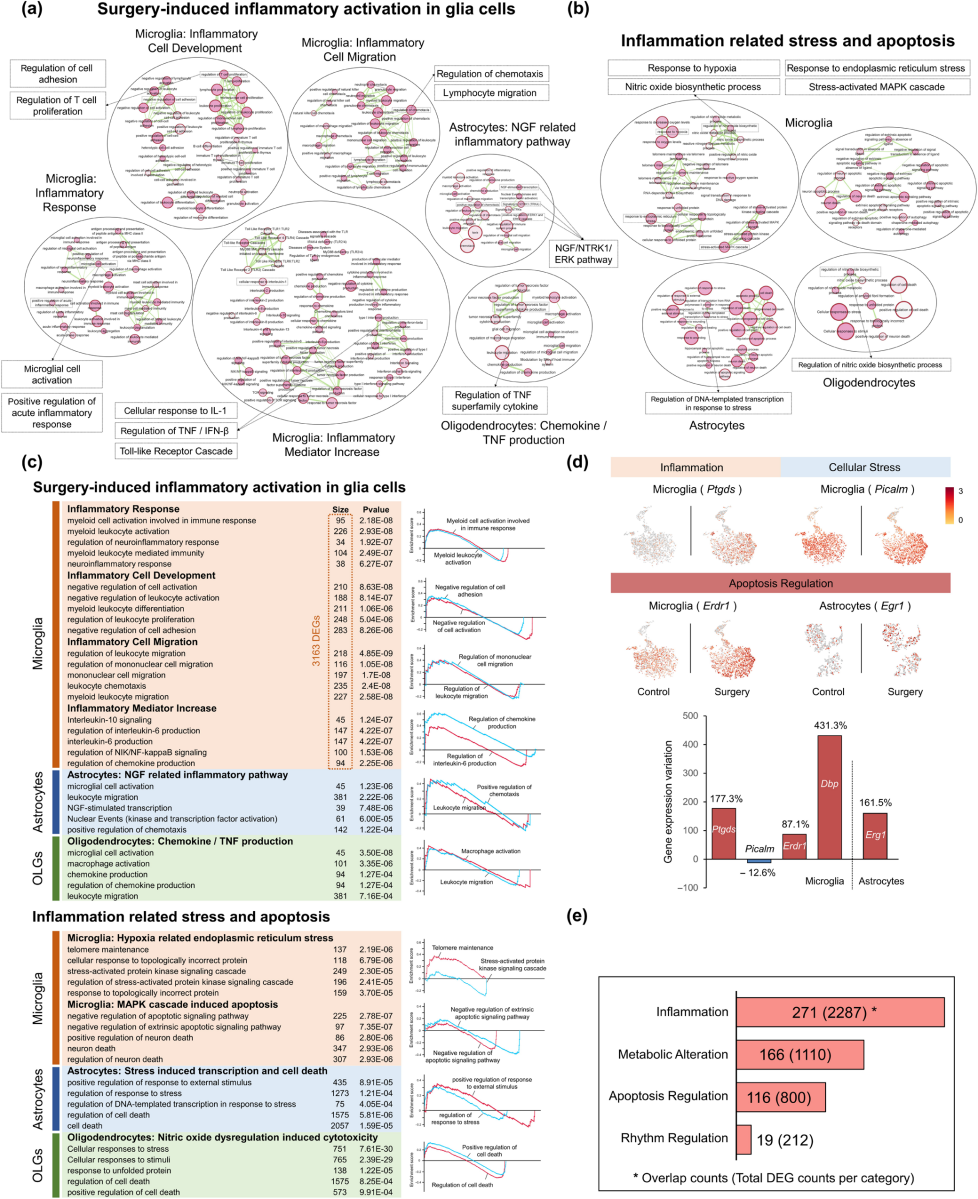
Activated microglia aggravates hippocampal inflammatory cytotoxicity and metabolic imbalance. (a) Surgery‐induced inflammatory activation in glia cells, which included four clusters of enriched terms in microglia, two clusters in astrocytes and oligodendrocytes. Other details are described in Figure 2a. (b) Inflammation related stress and apoptosis in glia cells. (c) The top 5 terms (ranked by *p* value) in each functional cluster showed in the chart with their DEGs counts (left). GSEA analysis of typical terms in each cluster (right). OLGs: Oligodendrocytes. (d) The expression profiles of DEGs from functional clusters and changes in gene expression during perioperative period. (e) Overlap analysis with Disease Associated Microglia (DAM) genes in AD (Keren‐Shaul et al., 2017). The bars represent the number of overlapping genes in each functional category, with the total number of genes in each category provided in parentheses.

Figure 3a showed surgery‐induced inflammatory activation in glia cells, which included four clusters of enriched terms in microglia and two clusters in astrocytes and oligodendrocytes. Figure 3c showed the top terms and their functional states assessed by GSEA analysis. The results indicated that the major terms were in microglia‐related clusters (3163 DEGs, Figure 3c). They included cluster Inflammatory Response with terms of microglial cell activation, and positive regulation of acute inflammatory response. The positive‐regulated ATP synthesis could meet the energy demand for these inflammatory responses (Figure 2e,f), which further affected cell adhesion and T cell proliferation, forming cluster Inflammatory Cell Development. The cluster Inflammatory Cell Migration included terms of immune cell chemotaxis and migration. Besides cell gathering terms, regulation of IL‐1/IL‐6, TNFs, IFN‐β, and Toll‐like receptor cascades were enriched in cluster Inflammatory Mediator Increase (Figure 3a), and DEG *Ptgds* (Figure 3d) was involved. Other glia cells also showed surgery‐induced inflammatory responses. Astrocytes included terms of NGF/NTRK1/ERK signaling pathway, which may result in astrocyte activation and cytokine/chemokine release. Oligodendrocytes included terms of TNF production. These inflammatory terms also interacted with cellular stress and apoptosis in microglia, astrocytes, and oligodendrocytes (Figure 3b,c) that included hypoxia response, endoplasmic reticulum stress from protein misfolding, and nitric oxide biosynthetic process (DEG *Picalm* was involved, Figure 3d). This could activate the MAPK cascade, and further regulate apoptotic signaling pathway (DEG *Erdr1* and *Egr1* were involved, Figure 3d). For the above 4 characteristic DEGs (*Ptgds*, *Picalm*, *Erdr1*, and *Egr1*), we showed their changes in the expression levels and distribution after surgery (Figure 3d, Figure S3e). *Picalm* decreased by 92.2%, while *Ptgds*, *Erdr1*, and *Egr1* increased by 177.3%, 87.1%, and 161.5%, respectively.

We further conducted a comparative analysis of DEGs in postoperative microglia and AD‐associated microglia (DAM, M1‐polarized state) (Keren‐Shaul et al., 2017). Remarkably, we found an overlap of 271 genes in inflammation cluster, 166 genes in metabolic alteration cluster, 116 genes in apoptosis regulation cluster, and 19 genes in rhythm regulation cluster (Figure 3e). These results suggested that postoperative microglia underwent the similar M1 polarization and metabolic imbalance with AD.

### 3.4 Cytotoxic astrocytes and oligodendrocytes disturb neuronal synaptic plasticity and axonal myelination

**FIGURE 4:**
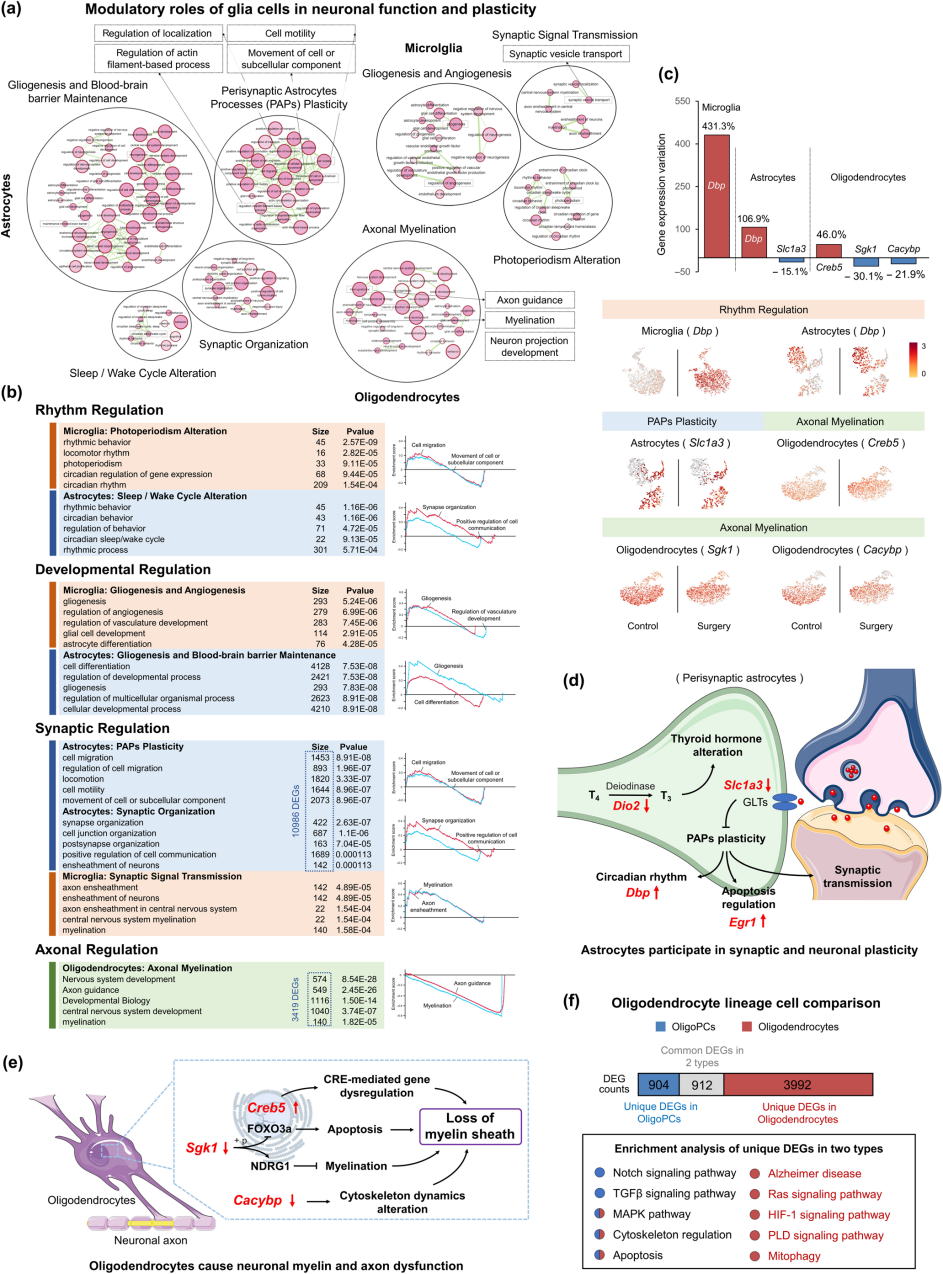
Cytotoxic astrocytes and oligodendrocytes disturb neuronal synaptic plasticity and axonal myelination. (a) Modulatory roles of glia cells in neuronal function and plasticity. Details are described in Figure 2a. (b) The top 5 terms (ranked by *p* value) in each functional cluster showed in the chart with their DEGs counts (left). GSEA analysis of typical terms in each cluster (right). (c) The expression profiles of DEGs from functional clusters and changes in gene expression during perioperative period. (d) Astrocytes participate in synaptic and neuronal plasticity. The red arrows indicate the direction of expression changes in DEGs. (e) Oligodendrocytes cause neuronal myelin and axon dysfunction. The red arrows indicate the direction of expression changes in DEGs. (f) Oligodendrocyte lineage cell comparison. The bar above represents DEG counts in oligoPCs and oligodendrocytes. The blue part indicates unique DEGs in oligoPCs, red for unique DEGs in oligodendrocytes, and gray for common DEGs in two types. The enrichment analysis of unique DEGs in two types is presented in the box below. The color of circles indicates the cellular source of term.

Figure 4a showed modulatory roles of microglia, astrocytes, and oligodendrocytes in neuronal function and plasticity, and Figure 4b showed top terms and their functional states assessed by GSEA analysis. Among them, microglia and astrocytes were involved in the rhythm regulation of neurons, which included terms of photoperiodism alterations, circadian sleep/wake cycle, and memory changes (DEG *Dbp* was involved, Figure 4c). The developmental terms in microglia included gliogenesis and angiogenesis (by VEGF production), while astrocytes included terms of neurogenesis, angiogenesis, and blood–brain barrier maintenance that based on glia‐vascular unit.

Astrocytes showed synaptic regulation on neurons through PAPs (Perisynaptic Astrocytes Processes) plasticity and synaptic organization. These synapse‐related clusters involved 10,986 DEGs (Figure 4b). Terms in cluster PAPs Plasticity included regulation of localization, cell motility, and subcellular component movement. These processes were related to the term of actin filament regulation, and possibly affected PAPs plasticity. It was crucial for synaptic function and neuroplasticity (DEG *Slc1a3* was involved, Figure 4c). Next, we used the above DEGs and pathways to hypothesize potential mechanisms involving perioperative astrocytes in the modulation of synaptic function (Figure 4d). *Dio2*, encoding a deiodinase, influences thyroid hormone metabolism by converting thyroxine (T4) to active triiodothyronine (T3) (Wei et al., 2023). Thyroid hormones can impact glucose metabolism, mitochondrial function, and overall energy production in astrocytes. Glutamate transporter (GLT), encoded by *Slc1a3*, is primarily expressed in astrocytes and is responsible for the uptake of glutamate from the synaptic cleft, contributing to the termination of synaptic transmission and preventing excitotoxicity (Pajarillo et al., 2019). During perioperative period, the reduced expression of *Slc1a3* may impair PAPs plasticity, consequently affecting circadian rhythm, apoptosis, and synaptic function in neurons (L. Wang et al., 2013).

Oligodendrocytes showed axonal regulation on neurons through myelination. This cluster included terms of axon guidance, myelination, and neuron projection development, which could be essential for morphology and function of neural networks. The protein metabolic alterations and energy deficits (described in Figure 2e–g) also indirectly influence myelination by affecting myelin proteins. We found 3419 DEGs in cluster Axonal Myelination (Figure 4b), and gene *Creb5*, *Sgk1*, and *Cacybp* were involved (Figure 4c). Next, we used the above DEGs to hypothesize potential mechanisms that oligodendrocytes cause neuronal myelin and axon dysfunction (Figure 4e). *Sgk1*, a kinase‐encoding gene, can phosphorylate FOXO3a and NDRG1 (Heller et al., 2014; Sahin et al., 2013). Postoperative decreased *Sgk1* enhances FOXO3a's capacity to activate pro‐apoptotic genes, leading to apoptosis and, indirectly, myelin loss. Additionally, reduced *Sgk1* can directly cause myelin loss by regulating NDRG1. The alteration of *Creb5* results in abnormal gene expression mediated by CRE, and *Cacybp* leads to cytoskeleton dynamics alteration. Both factors may impact the regulation of oligodendrocytes in the neuronal myelination. Subsequently, we conducted a comparative analysis of oligodendrocytes and oligoPCs (Figure 4f). The results showed that 912 genes exhibited altered expression in both cell types, with 904 genes exclusively changing in oligoPCs and 3992 genes exclusively changing in oligodendrocytes (genes with Bonferroni‐corrected *p* value <0.05). We then performed enrichment analysis on the unique DEGs in each cell type. For oligodendrocytes, alterations in phospholipase D (PLD) and HIF‐1 signaling pathways indicated other potential causes of myelin sheath loss described in Figure 4e. The Ras signaling pathway can cause nitric oxide dysregulation, which impair astrocyte endfeet and blood–brain barrier maintenance (shown in Gliogenesis and Blood–brain barrier Maintenance, Figure 4a), and promote apoptosis in neurons (shown in Neurotoxicity, Figure 2a). The results also showed degeneration features of AD.

For this part, we found five characteristic DEGs (*Dbp*, *Slc1a3*, *Creb5*, *Sgk1*, and *Cacybp*) in microglia, astrocytes, and oligodendrocytes, and showed their changes in the expression levels and distribution after surgery (Figure 4c, Figure S3e). *Dbp* increased by 431.3% and 106.9% in microglia and astrocytes, respectively. *Slc1a3*, *Sgk1*, and *Cacybp* decreased by 15.1%, 30.1% and 21.9%, while *Creb5* increased by 46.0%.

### 3.5 Ligand‐receptor communication in glia–neuron cycle provokes neuroinflammation and disturbance in synaptic/myelin dynamics

**FIGURE 5:**
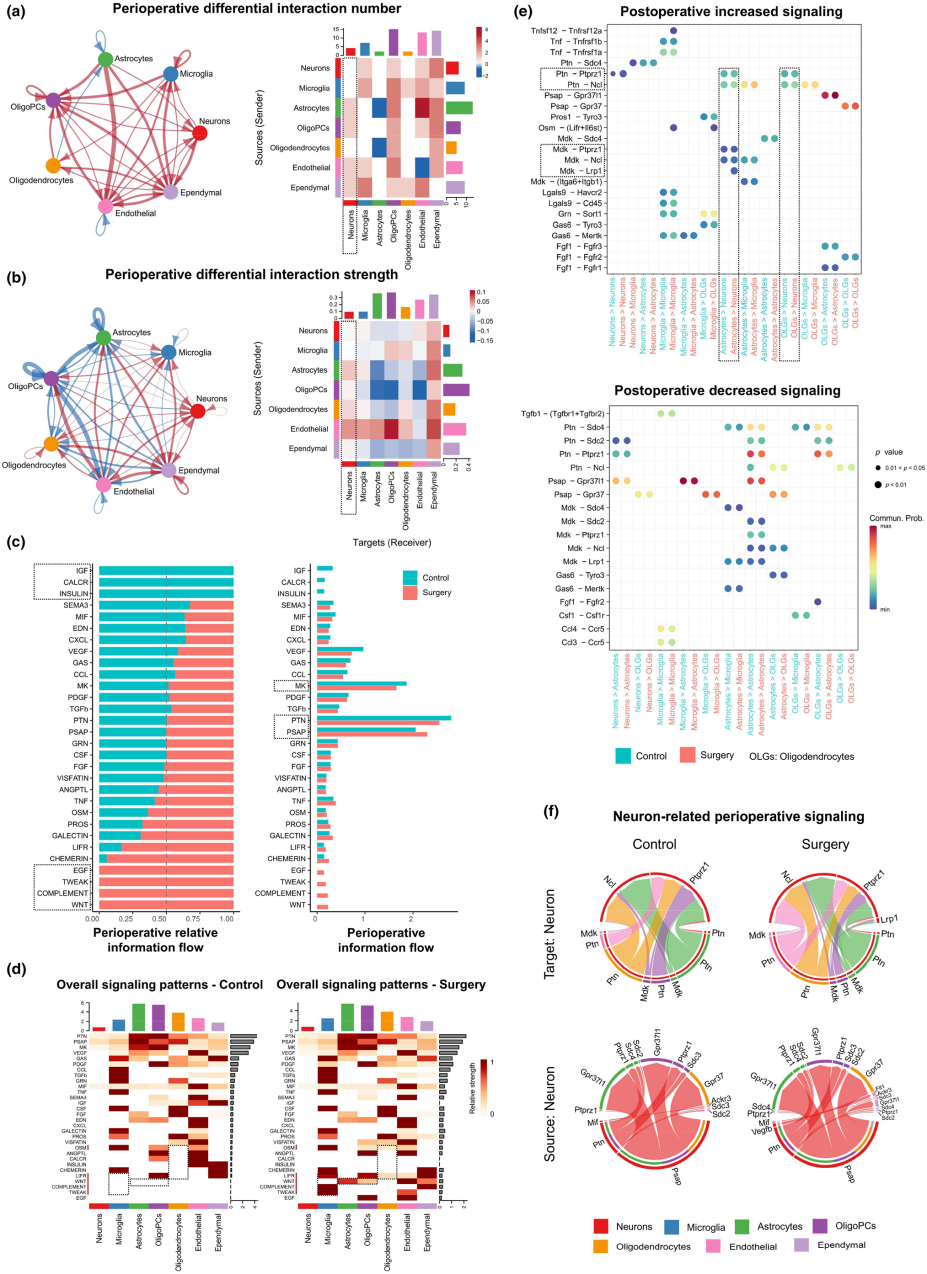
Ligand‐receptor communication in glia–neuron provokes neuroinflammation and disturbance in synaptic/myelin dynamics. (a,b) Circle plots and heatmaps of perioperative differential interaction quantity and strength between seven cell types in aged hippocampus. Blue lines/rectangles indicate that the displayed communication decreases after surgery, whereas red lines/rectangles indicate increase. The bars at the top (side) of heatmaps represent the overall levels of alterations for each cell type as targets (sources). (c) Bar plots of the ranking of signaling pathways by overall information flow differences between control group and surgery group. The top signaling pathways are more enriched in control group, the middle ones are equally enriched, and the bottom ones are more enriched in surgery group. (d) Heatmaps of overall (comprising both outgoing and incoming) signaling flow patterns of each cell type mediated by individual signaling pathway in control group and surgery group. (e) Comparison of the significant ligand‐receptor pairs from neuron and three types of glia cell between control and surgery groups. Dot color reflects communication probabilities and dot size represents computed *p* values. Empty space means the communication probability is zero. *p* values are computed from one‐sided permutation test. OLGs: Oligodendrocytes. (f) Neuron‐related perioperative signaling in control and surgery groups.

We applied CellChat to reveal perioperative intercellular signaling communications. The results indicated the number of ligand‐receptor pairs mainly increased after surgery (red lines and rectangles, Figure 5a). Among them, 4 total pairs targeting neurons increased from astrocytes, oligoPCs, endothelial, and ependymal (Figure 5a, right). The most increased interactions were observed sourced from astrocytes (11 pairs increased). Interactions from microglia to oligodendrocytes also increased by 1 pair. We also found an overall increase in the strength of interactions (ligand‐receptor communication probability) targeting neurons (Figure 5b, left), which totally increased by 0.1 (Figure 5b, right). Interaction strength from microglia to oligodendrocytes also increased by 0.02. These results indicated that interactions targeting neurons increased both in quantity and strength.

To dissect the above changes, we calculated the information flows for each signaling pathway (Figure 5c), which is defined as total communication probability among all cell types (Jin et al., 2021). We found that IGF, CALCR, and INSULIN signaling pathways turned off while the WNT, COMPLEMENT, TWEAK, and EGF pathways turned on after surgery (Figure 5c, left). PTN, MK, and PSAP pathways showed the largest information flow (Figure 5c, right). We then distinguished these signaling pathways within specific cell type (Figure 5d). LIFR, COMPLEMENT, and TWEAK increased in microglia. WNT increased in astrocytes and oligoPCs. OSM and LIFR increased in oligodendrocytes. COMPLEMENT pathway produced C3a and C5a, causing microglia activation (shown in cluster Microglia: Inflammatory Response, Figure 3a). TWEAK is a member of TNF superfamily (shown in cluster Microglia: Inflammatory Mediator Increase), and can induce apoptosis in central nervous system with help of downstream Fn14 (shown in cluster Stress and Apoptosis, Figure 3b). OSM pathway can be activated in demyelinated regions and mediate the repairment, reflecting the occurrence of postoperative myelin loss (shown in Figure 4e).

Then, we analyzed the alterations of ligand‐receptor pairs between glia cells and neurons (Figure 5e,f). The results indicated that astrocytes and oligodendrocytes mainly interacted with neurons, and these ligand‐receptor communications were enhanced after surgery (Figure 5e, upper part). Among them, the ligand MDK and its receptor Lrp1, Ptprz1, and Ncl were major increasing pathways from astrocytes to neurons. Enhanced MDK‐Lrp1 can impair synaptic transmission by altering the expression of GLTs, which may closely relate to the DEG *Slc1a3* (shown in Figure 4d). Lrp1 is also a neuronal receptor for α‐synuclein uptake and spread (Chen et al., 2022), and further causes neurotoxicity (shown in Figure 2a). This validated our previous functional prediction of Lrp1 (Suo et al., 2022). Besides, Ligand PTN and its receptor Ptprz1 and Ncl were also increasing from both astrocytes and oligodendrocytes to neurons. PTN, by regulating Ptprz1, plays a crucial role in oligodendrocyte differentiation and remyelination in response to demyelinating events (Kuboyama et al., 2015). Enhanced PTN‐Ptprz1 further suggested the myelin loss after surgery, and may closely relate to the myelin DEGs (shown in Figure 4e). Together, CellChat's joint analysis indicated that ligand‐receptor communication in glia–neuron provokes neuroinflammation and disturbance in synaptic/myelin dynamics. It should be noted that this represents just one aspect of how glia cells influence neurons (Figure 5f). Establishing a more comprehensive pathological network is necessary to understand the transcellular pathogenesis of postoperative cognitive impairment.

### 3.6 The integrated neuroglial system contributes to postoperative cognitive impairment

**FIGURE 6:**
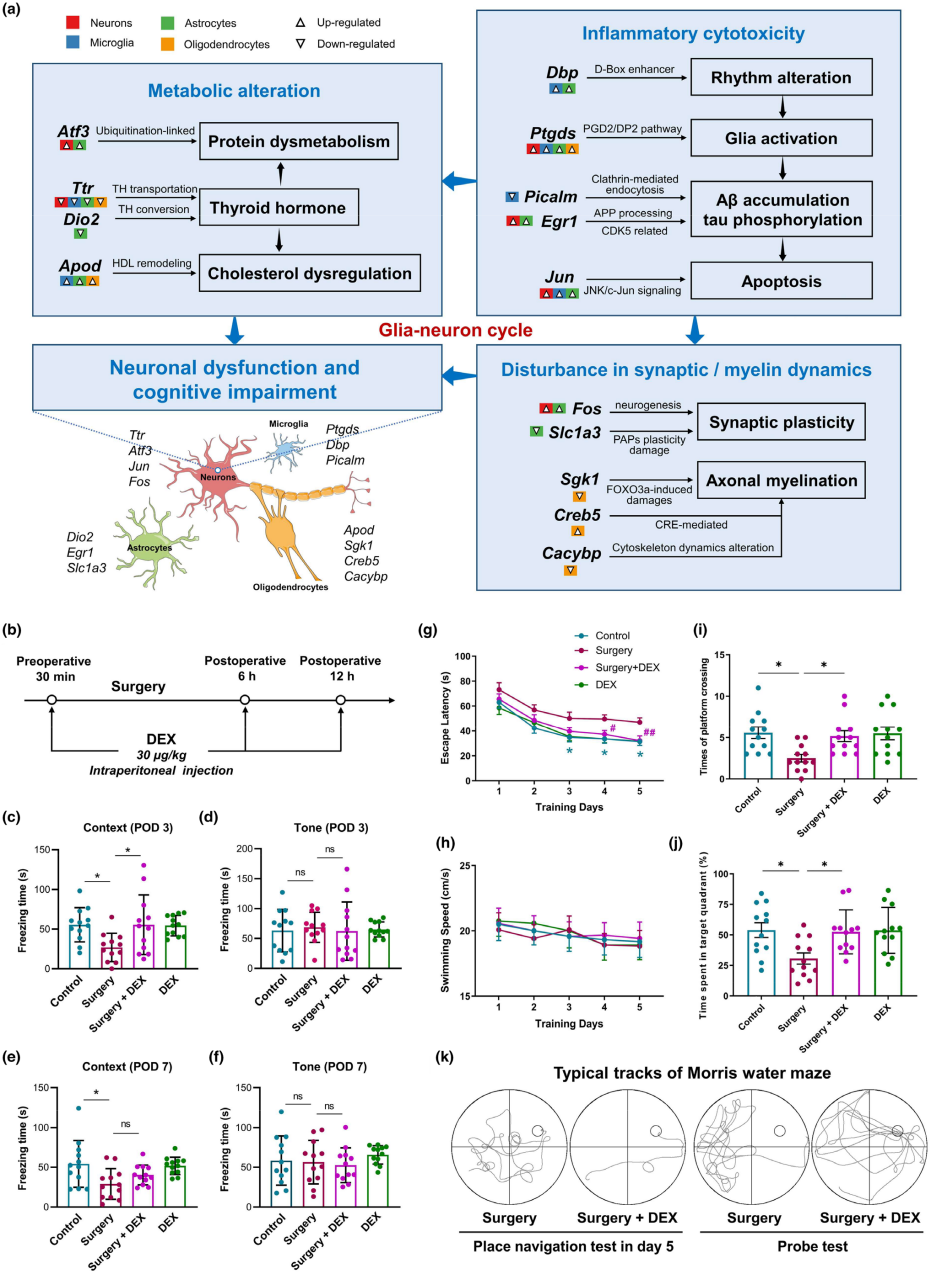
The integrated neuroglial system contributes to postoperative cognitive impairment. (a) Each square beneath every gene represents a cell type, with internal arrows indicating upregulation or downregulation of the gene. (b) Schematic of experimental approach for DEX treatment. (c, e) In context test, the freezing time decreased significantly in surgery group compared with control group at POD 3 and POD 7. DEX specifically increased the freezing time only at POD 3. (d, f) In tone test, there was no significant between two groups at POD 3 or POD 7. (g) Morris water maze showed that in the place navigation test, the escape latency increased significantly in surgery group compared with control group at POD 3, POD 4, and POD 5, and DEX rescued this surgery‐induced effect at POD 4 and POD 5. (h) The swimming speed did not change significantly in four groups. (i, j) In the probe test, both the times of platform crossing and the time spent in target quadrant decreased significantly after surgery, and increased again in DEX group. (k) The typical tracks of Morris water maze. Means ± SD, *p < 0.05, **p < 0.01, ns: Not significant.

Based on gene enrichment and cell communication results, we developed an integrated neuroglial system emphasizing perioperative interactions between microglia, astrocytes, oligodendrocytes, and neurons (Figure 6a). This system primarily included metabolic alteration, inflammatory cytotoxicity, and disturbance in synaptic/myelin dynamics. We elucidated the genetic underpinnings of these three major pathological parts, along with the expression profiles of relevant 14 DEGs across neuron and glia cells.

The neuroglial system underwent widespread metabolic alterations after surgery, including ubiquitination‐linked protein dysmetabolism mediated by stress‐responsive gene *Atf3* (increased in neurons and astrocytes), cholesterol dysregulation mediated by *Apod* (increased in microglia, astrocytes, and oligodendrocytes) through HDL composition (Pedrini et al., 2022), and thyroid hormone abnormalities that may regulate both. *Ttr*, encoding transthyretin, can facilitate thyroid hormone transport across the blood‐cerebrospinal fluid barrier (Gião et al., 2020). Decreased *Ttr* in all 4 cell types, along with reduced *Dio2* (described in Figure 4d) in astrocytes, may both contribute to dysregulated thyroid hormone levels. Inflammatory cytotoxicity can aggravate the above metabolic changes. The rhythm alteration mediated by clock gene *Dbp* (increased in microglia and astrocytes) can affect the fluctuation of glia activation. This inflammatory response was mediated by *Ptgds* (increased in all 4 cell types), which can synthesize prostaglandin D2 (PGD2) and contribute to PGD2/DP2 pathway (Corwin et al., 2018). Glia activation also amplified cytotoxicity in conjunction with Aβ accumulation, tau phosphorylation, and apoptosis. *Picalm*, crucial for clathrin‐mediated endocytosis and autophagy (Ando et al., 2020), decreased only in microglia and led to Aβ accumulation. *Egr1* (increased in neurons and astrocytes) modulated tau phosphorylation and Aβ synthesis by regulating CDK5 and amyloid precursor protein metabolites (Qin et al., 2017). *Jun* (increased in neurons, microglia, and astrocytes) regulated apoptosis through JNK/c‐Jun signaling. The increased inflammatory cytotoxicity further caused disturbance in synaptic/myelin dynamics, including *Fos* (decreased in neurons and astrocytes) mediated cellular suppression following surgical inflammation and stress, *Slc1a3* (decreased in astrocytes, described in Figure 4d) mediated PAPs plasticity damage, and oligodendrocytes induced myelin dysfunction (*Sgk1*, *Creb5*, and *Cacybp*, described in Figure 4e). The above disrupted glia–neuron cycle eventually caused neuronal dysfunction and subsequent postoperative cognitive impairment.

To verify the expression of above mentioned 14 key genes within specific cell type during perioperative period and further validate the results of scRNA‐seq, we performed RNA FISH. Based on the results of differentially expressed analysis and gene function, we determined the validation cell types for these 14 genes. Neurons (marked by Map2) included *Ttr*, *Atf3*, *Jun*, and *Fos*; microglia (marked by Iba1) included *Ptgds*, *Dbp*, and *Picalm*; astrocytes (marked by Gfap) included *Dio2*, *Egr1*, and *Slc1a3*; oligodendrocytes (marked by Mbp) included *Apod*, *Sgk1*, *Cacybp*, and *Creb5*. The FISH results indicated that, following standardized surgery, 8 genes (*Ptgds*, *Dbp*, *Egr1*, *Apod*, *Creb5*, *Atf3*, *Jun*, and *Fos*) in specific cell type increased, and 6 genes (*Picalm*, *Dio2*, *Slc1a3*, *Sgk1*, *Cacybp*, and *Ttr*) decreased, which was consistent with the results of scRNA‐seq (Figures S4 and S5).

### 3.7 DEX alleviates cognitive impairment by targeting protein dysmetabolism in disrupted glia–neuron cycle

Based on the disrupted glia–neuron cycle, we established a perioperative DEX intervention model. DEX was administered at a dose of 30 μg/kg via intraperitoneal injection 30 min before surgery, as well as 6 and 12 h after surgery (Figure 6b). Combined with previous studies and our behavioral preliminary experiments, we set the above dosage (Mei et al., 2021). This stimulated the clinical DEX combined anesthesia and analgesia protocol, which can reduce the dosage of anesthetic sevoflurane. Fear condition test indicated hippocampus‐dependent (Context) cognition impairment at 3 days (two‐way ANOVA followed by Bonferroni *post‐hoc* test, *p* = 0.0362, Figure 6c) and 7 days (two‐way ANOVA followed by Bonferroni *post‐hoc* test, *p* = 0.0162, Figure 6e) after surgery. These impairments reflected the poor retention of learning content during training, which was closely related to gene expression changes 1 day after surgery. Hippocampus‐independent (Tone) cognition showed no significant changes (*p* > 0.05, Figure 6d,f). Moreover, DEX improved hippocampus‐dependent cognition 3 days after surgery (two‐way ANOVA followed by Bonferroni *post‐hoc* test, *p* = 0.0364, Figure 6c). Morris water maze indicated that navigation escape latency decreased significantly with ongoing training in control group, and this trend appeared less pronounced in surgery group, which can be improved by DEX (Figure 6g). The memory ability, assessed by platform crossing and target quadrant stay, impaired after surgery (two‐way ANOVA followed by Bonferroni *post‐hoc* test, *p* = 0.0111, *p* = 0.0212, Figure 6i,j), which can be rescued by DEX intervention (two‐way ANOVA followed by Bonferroni *post‐hoc* test, *p* = 0.0381, *p* = 0.0355, Figure 6i,j). Based on the above results of fear condition test and Morris water maze, we hypothesized that the improvement of postoperative cognitive impairment by DEX may be achieved by acting on the glia–neuron cycle.

**FIGURE 7:**
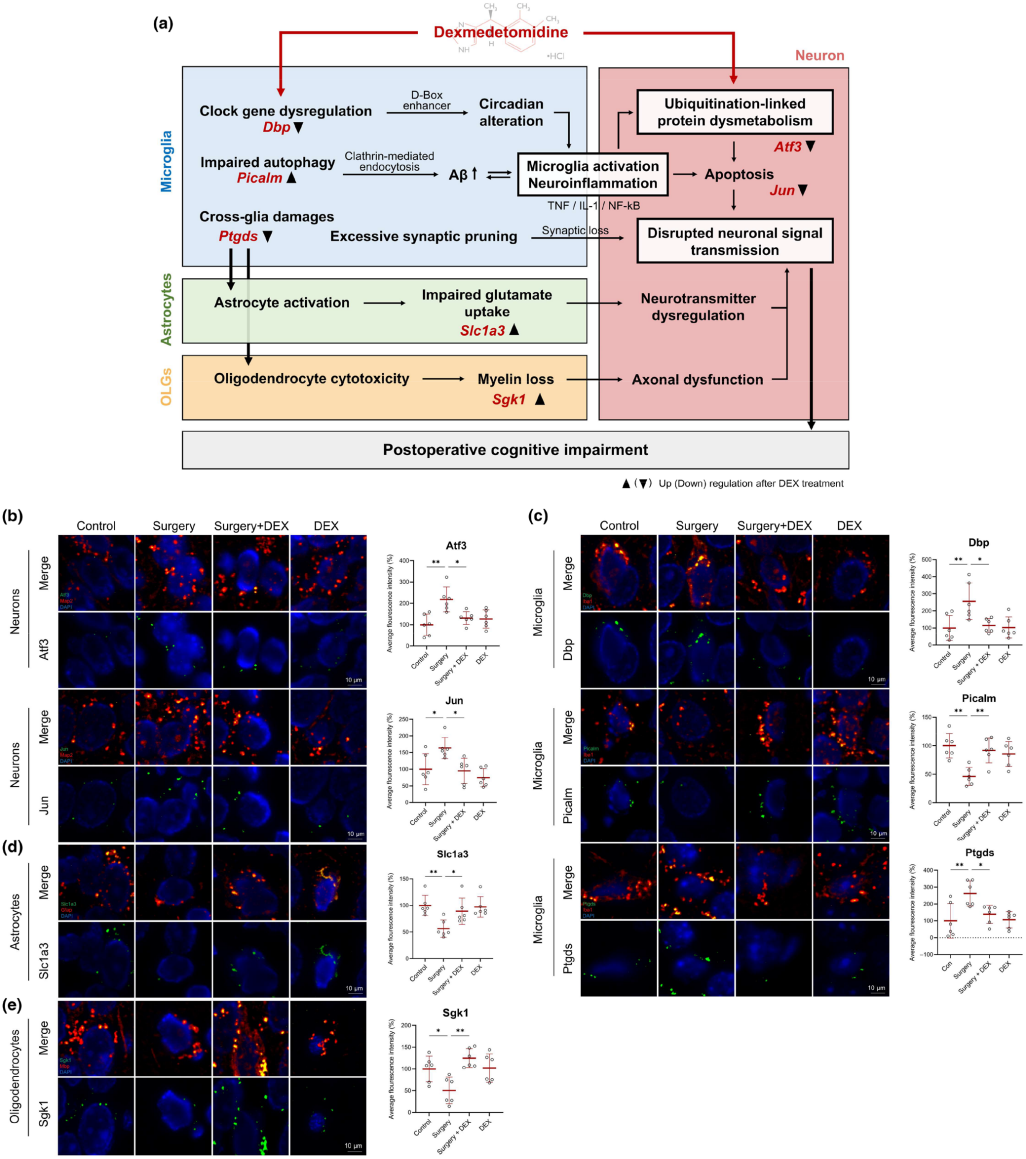
DEX alleviates cognitive impairment by targeting protein dysmetabolism in disrupted glia–neuron cycle. (a) The candidate therapeutic network of DEX. The black triangular arrows show up/down regulation of seven genes after DEX treatment. OLGs: Oligodendrocytes. (b–e) RNA FISH of seven altered key genes in perioperative neurons, microglia, astrocytes, and oligodendrocytes under the DEX intervention. The positive *Atf3* and *Jun* mRNA hybridization (green) were observed in Map2 positive cells (red). The positive *Ptgds*, *Dbp* and *Picalm* mRNA hybridization (green) were observed in Iba‐1 positive cells (red). The positive *Slc1a3* mRNA hybridization (green) was observed in Gfap positive cells (red). The positive *Sgk1* mRNA hybridization (green) was observed in Mbp positive cells (red). DAPI (blue) was used as a counterstaining to show nuclei. Scale bar = 10 μm. The quantification graphs on the right show the average fluorescence intensity of each gene. Means ± SD, *p < 0.05, **p < 0.01 and ***p < 0.001.

The neuroglial system depicted in Figure 6a revealed potential pathological changes associated with postoperative cognitive impairment and identified 14 key DEGs responsible for these alterations. To explore how DEX alleviates cognitive impairment, we conducted RNA FISH on these key genes and observed the reversal of seven genes in glia–neuron cycle under DEX intervention. Based on these findings, we established a candidate therapeutic network (Figure 7a). DEX targeted ubiquitination‐linked protein dysmetabolism in neurons (*Atf3* decreased, Figure 7b) and ameliorated apoptosis (*Jun* decreased, Figure 7b). It also alleviated clock gene dysregulation (*Dbp* decreased, Figure 7c) and impaired autophagy (*Picalm* increased, Figure 7c). These effects reduced inflammatory gene activation and Aβ aggregation, therefore mitigating microglia activation and neuroinflammation. The reduced neuroinflammation further ameliorated protein dysmetabolism and apoptosis. Inhibited microglia activation reduced excessive synaptic pruning and prevented synaptic loss. This microglia inhibition also suppressed cross‐glia damages in astrocyte activation and oligodendrocyte cytotoxicity (*Ptgds* decreased, Figure 7c). The former ameliorated impaired glutamate uptake (*Slc1a3* increased, Figure 7d), thereby addressing neurotransmitter dysregulation. The latter mitigated myelin loss (*Sgk1* increased, Figure 7e), thus improving axonal dysfunction in neurons. Together, these DEX‐mediated effects enhanced neuronal signal transmission and synaptic function, and further mitigated postoperative cognitive impairment.

## 4 DISCUSSION

The regulatory effects of microglia, astrocytes, and oligodendrocytes on neuronal function involve multiple targets, and abnormal interactions between these cells contribute to aging‐related postoperative cognitive impairment. However, there are few studies on this dysregulated glia–neuron cycle. Herein, we performed single‐cell sequencing to explore the hippocampal overall landscape. We found neurons underwent widespread metabolic alterations and mitochondrial injuries after surgery, with the highest proportion of up‐regulated genes (ratio = 19.71). Protein catabolic process was also considered as the possible neuronal intervention targets of DEX revealed by DrugBank database (described in Figure 2d). We then scrutinized the disrupted glia–neuron cycle responsible for the above neuronal dysfunction. Postoperative microglia underwent activation that observed in AD (described in Figure 3e), and aggravated inflammatory cytotoxicity in the neuroglial system. Cytotoxic astrocytes impaired neuronal synaptic function via “tripartite synapses” model after surgery (described in Figure 4d), while oligodendrocytes affected the myelination of neuronal axons, and thereby affecting the neuronal signal transmission (described in Figure 4e).

Our results indicated that impaired circadian rhythm (dysregulated clock gene *Dbp*, Figure 4c) and disabled phagocytosis for Aβ clearing (dysregulated gene *Picalm*, Figure 3d) led to microglia activation and neuroinflammation. We found inflammatory cytokines (such as TNF‐α, IL‐1, IL‐6, Figure 3a) activated the PI3K/Akt/mTOR pathway, which may enhance HIF‐1α expression in neurons and cause stress‐related metabolic reprogramming from oxidative phosphorylation to glycolysis (Murai & Matsuda, 2023; Pan et al., 2022). This shift led to insufficient cellular energy supply and caused ATP‐dependent protein catabolic dysregulation, which affected a molecular chaperone protein TRiC after surgery (described in Figure 2a). Dysregulated TRiC may impair axonal BDNF and lysosomal transport, and further induce neuronal apoptosis. Previous studies suggested that TRiC reagent‐mediated reductions in mutant huntingtin enhanced BDNF delivery, thereby restoring the trophic status of neurons (Zhao et al., 2016). The above neuronal dysmetabolism also caused neurotoxicity by increasing oxidative stress in neuronal mitochondria and endoplasmic reticulum (dysregulated *Jun*, Figure 2c). This may trigger neuronal damages through protein folding disturbances (Ashleigh et al., 2023; Lim et al., 2023).

Postoperative microglia affected astrocytes through PGD2 and complement pathways in a “tripartite synapses” model that formed by astrocyte processes and neuronal synapses (Figure 4d). PGD2 (synthesized by *ptgds*) is produced by reactive microglia and acts as a key inflammatory mediator. It binds to DP receptors and triggers the transition of astrocytes to harmful A1 phenotype, which causes increased production of GFAP, local blood flow enhancement, enhanced antigen presentation, and the release of chemotactic factors (Mohri et al., 2006). Microglia‐induced complement C3^+^ astrocytes (COMPLEMENT pathway was switched on, Figure 5d) also exhibit reduced synaptogenic factors and phagocytosis receptors (Lee et al., 2021; Singh et al., 2016), impairing astrocyte‐driven synaptogenesis and synaptic pruning. The impaired astrocytes described above exhibited dysregulation of GLT (encoded by *slc1a3*, Figure 4c), which affected glutamate level in synaptic cleft and subsequent synaptic transmission after surgery. These pathological changes are also seen in multiple sclerosis and Parkinson's disease (Booth et al., 2017), resulting in subsequent excitotoxic neuronal death. Disease‐associated microglia can target nearby myelin damage to phagocytize amyloid plaques, causing oligodendrocyte cytotoxicity (Safaiyan et al., 2016). Impaired oligodendrocytes affected the structural integrity of myelin sheaths in neurons, which could impair the axonal function (dysregulated *Cacybp*, *Sgk1*, and *Creb5*, Figure 4c). The above‐mentioned synaptic dysfunction and axonal impairments both disrupt neuronal communication in aged hippocampus, leading to memory loss and cognitive impairment. Our results also showed a reduction in the number of ependymal cells in the surgery group, and this difference was consistent across samples, not driven by any individual sample. Gene expression analysis confirmed a high correlation (0.97, Figure S2e) between the control and surgery groups, with stable expression patterns across all samples. Our data underwent strict quality control procedures (details in methods), ensuring its reliability. Additionally, similar reductions in ependymal cells have been reported in AD and aging models (Allen et al., 2023; Habib et al., 2020).

## 5 CONCLUSION

In this study, we presented an aged hippocampal single‐cell atlas and examined the role of disrupted glia–neuron cycle in postoperative cognitive impairment. Postoperative neurons and glia exhibited widespread protein dysmetabolism and mitochondrial electron misrouting, which was exacerbated by microglia activation/neuroinflammation. Reactive microglia also aggravated astrocyte and oligodendrocyte cytotoxicity, altering glutamate level and synaptic function, and affecting neuronal myelination. The therapeutic network of DEX targeted neuronal metabolic imbalance and boosted signal transmission. These findings deepen our understanding of the neurobiological basis of postoperative cognitive impairment in the elderly population and highlight the potential of glia–neuron cycle targeted therapy for prevention.

## AUTHOR CONTRIBUTIONS

Z.S. analyzed the data, performed cross‐cellular function analysis, validation, behavior experiments, and wrote the manuscript. T.X. contributed to the study design, data analysis and manuscript revision. Y.Q. constructed animal model, performed single‐cell experiments and gene function analysis, and contributed to manuscript revision. Y.Z. and W.X. contributed to animal experiments, tissue collection, and data analysis. B.Z. contributed to study design and data analysis. J.Y. (J. Yang) and J.Y. (J. Yu) contributed to data analysis and manuscript revision. H.Z. contributed to study design, supervision, and manuscript revision. C.N. designed and supervised the project, contributed to the analysis and interpretation of data, provided the funding support, wrote, and revised the manuscript. All authors read and approved the final manuscript.

## FUNDING INFORMATION

This work was supported by the National Natural Science Foundation of China (No. 82171195), Nonprofit Central Research Institute Fund of Chinese Academy of Medical Sciences (No. 2023‐JKCS‐25), National Key R&D Program of China (No. 2022YFF0705004), Beijing Natural Science Foundation (No. 7232131), and Talent Project of National Cancer Center/Cancer Hospital Chinese Academy of Medical Sciences (For Dr. Cheng Ni).

## CONFLICT OF INTEREST STATEMENT

The authors declare no conflicts of interest.

## Supporting information


Figures S1–S5

Tables S1–S2

## Data Availability

scRNA‐seq data presented in this study are available in the Gene Expression Omnibus (GEO) with accession number GSE267933. The other data that support the findings of the study are available from the corresponding author upon reasonable request.
