# Supplementary material for: Aged hippocampal single‐cell atlas screening unveils disrupted neuroglial system in postoperative cognitive impairment
Source: Aging Cell. 2024 Nov 14;24(3):e14406. doi: 10.1111/acel.14406 (PMC11896209; doi:10.1111/acel.14406)
Supplement: Supplementary file 1 — Figures S1–S5 [file ACEL-24-e14406-s002.pdf]

FIGURE S1

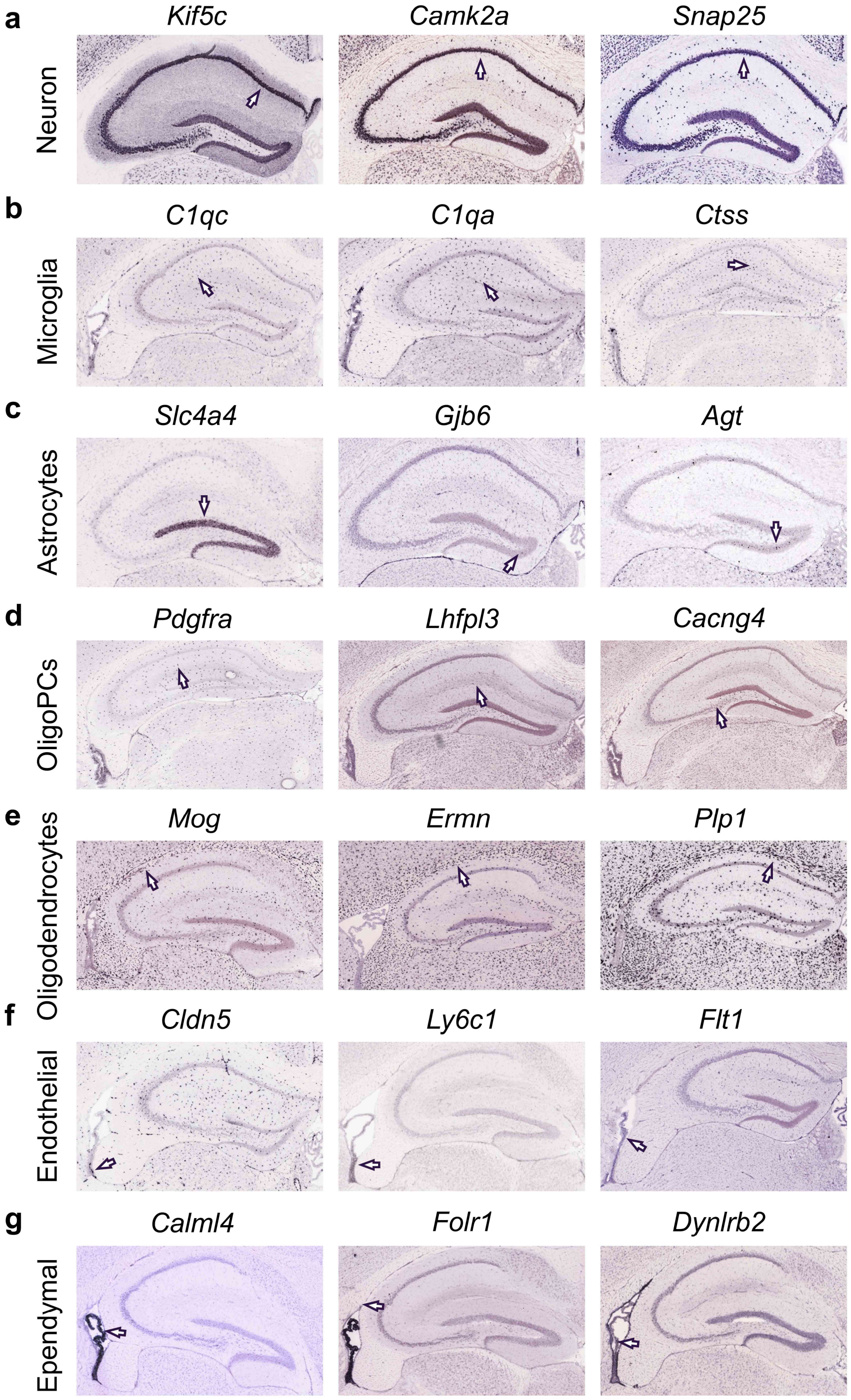

Cross-validation of marker genes for 7 major cell types. To validate the specificity of marker genes and show the spatial distribution of 7 major cell types, the expression of cell markers was examined in the in situ hybridization images from the Allen Brain Atlas (Lein et al., 2007).

FIGURE S2

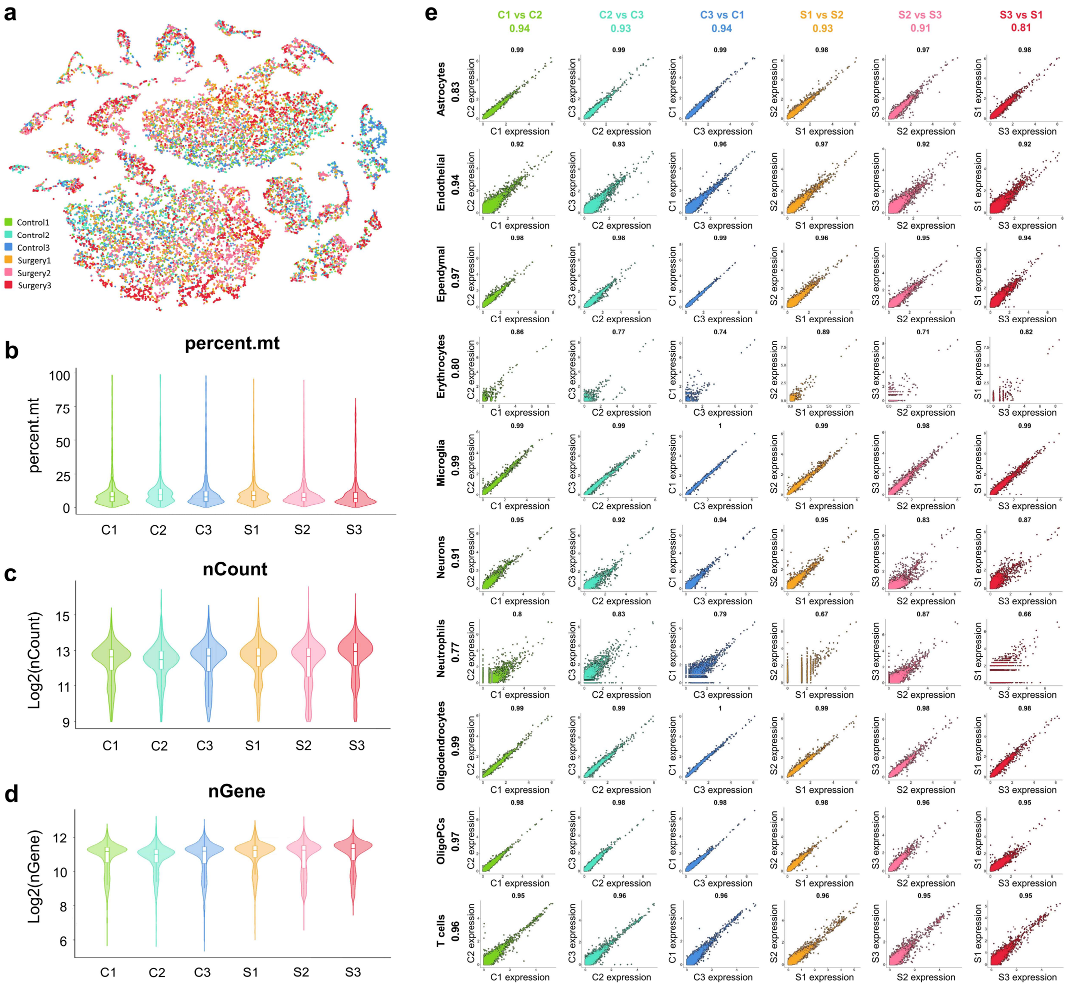

Drop-seq library statistics and quality control (QC) features. (a) Single cells were colored by sample origin (3 control and 3 surgery group animals) on the t-SNE plot. None of the clusters were driven exclusively by a sample, as single cells from all samples are present in each cluster. We do, however, see segregation within some of the clusters due to surgery vs. control status. (b-d) scRNA-seq data quality control. (e) Within- and between-group gene-gene correlations for each cell type identified indicate overall coherence of gene expression between samples. Gene-gene correlations between pairwise combinations of samples (both between and within group) were assessed for each cell type. Numbers within the individual plots indicate the correlation of gene expression levels between the two indicated samples for the specified cell type. The number next to the cell type labels on the left side of the plots indicates the average between-group correlation (Control vs Surgery). The number on the top of each column indicates the average correlation between the two indicated samples across all cell types.

FIGURE S3

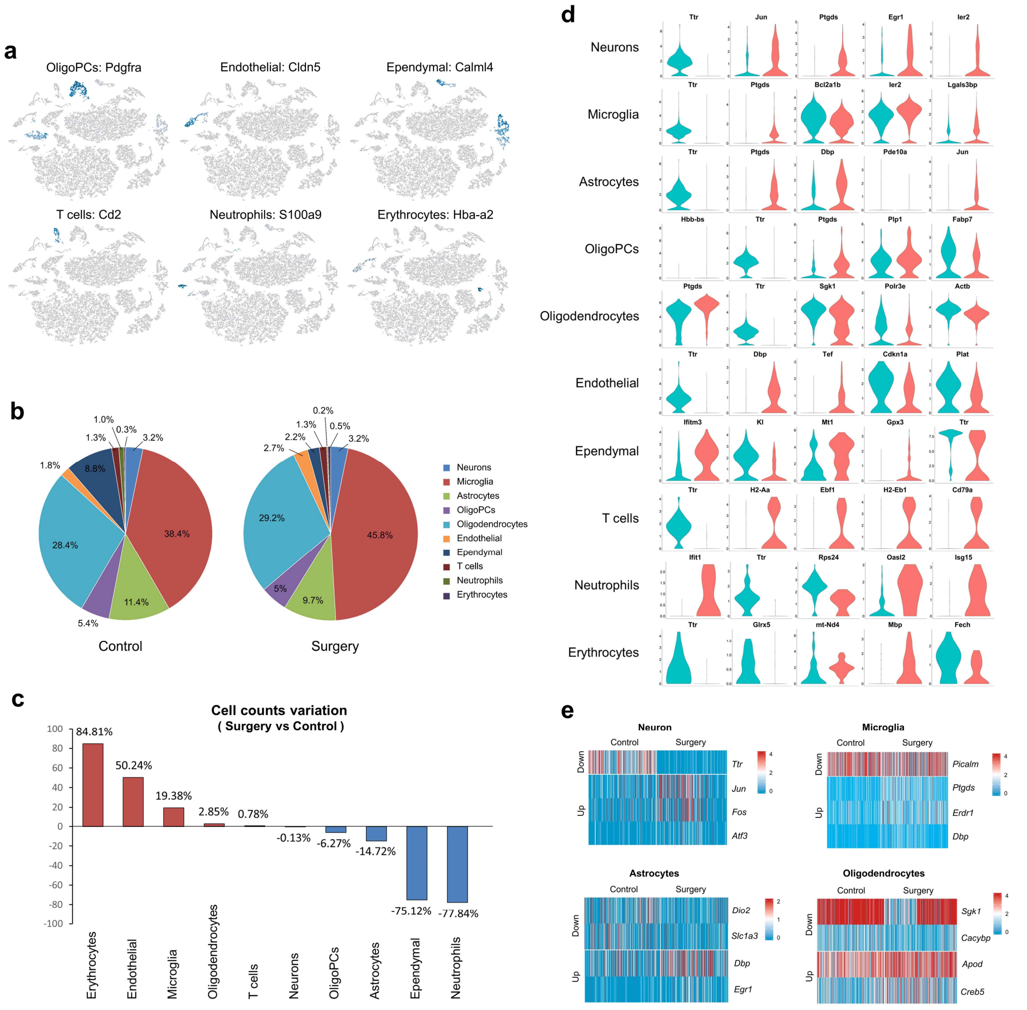

Widespread alterations in aged hippocampal cell types after surgery. (a) Cluster specific expression of cell-type specific markers: Pdgfra for oligoPCs, Cldn5 for endothelial, Calml4 for ependymal, Cd2 for T cells, S100a9 for neutrophils, and Hba-a2 for Erythrocytes. (b) The cell population proportions between control group and surgery group. (c) Corresponding counts variation in 10 cell types (increase in red and decrease in blue). (d) The expression level of top 5 DEGs shown in the violin plot (control group in cyan and surgery group in pink). (e) The heatmaps illustrate changes in the expression levels of characteristic DEGs after surgery.

FIGURE S4

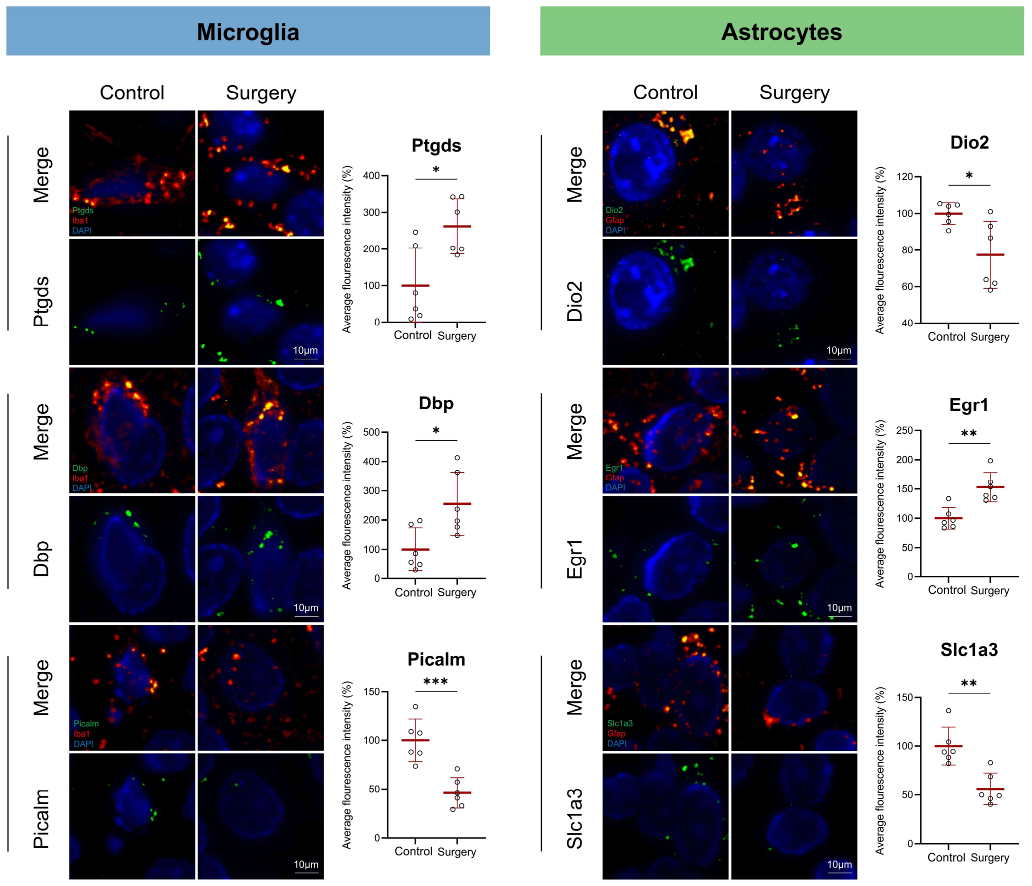

RNA FISH of 3 DEGs in microglia and 3 DEGs in astrocytes during perioperative period. The positive *Ptgds*, *Dbp* and *Picalm* mRNA hybridization (green) were observed in Iba-1 positive cells (red). The positive *Dio2*, *Egr1* and *Slc1a3* mRNA hybridization (green) were observed in Gfap positive cells (red). DAPI (blue) was used as a counterstaining to show nuclei. Scale bar = 10 μm. The quantification graphs on the right show the average fluorescence intensity of each gene. Means ± SD, *$p < 0.05$, **$p < 0.01$ and ***$p < 0.001$.

FIGURE S5

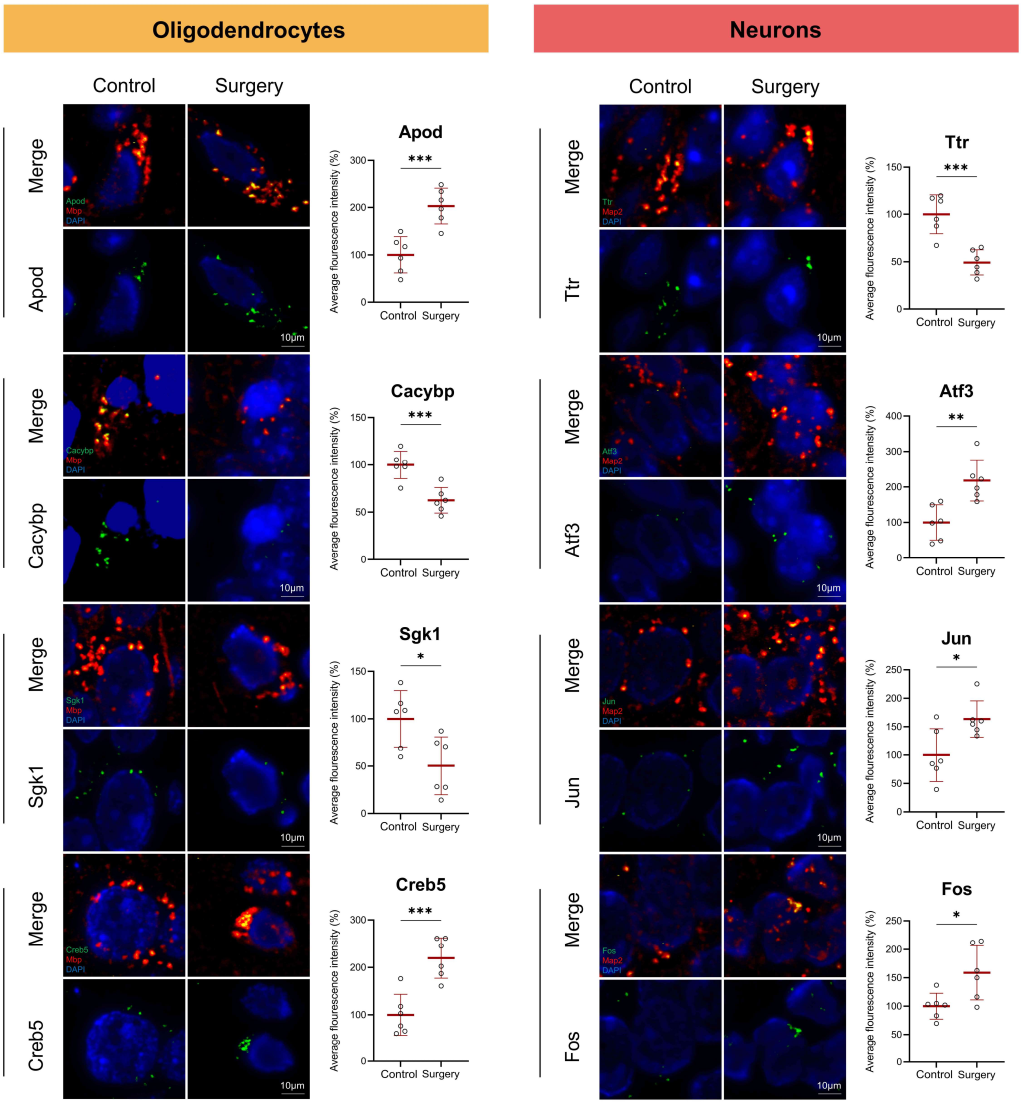

RNA FISH of 4 DEGs in oligodendrocytes and 4 DEGs in neurons during perioperative period. The positive *Apod*, *Cacybp*, *Sgk1*, and *Creb5* mRNA hybridization (green) were observed in Mbp positive cells (red). The positive *Ttr*, *Atf3*, *Jun* and *Fos* mRNA hybridization (green) were observed in Map2 positive cells (red). DAPI (blue) was used as a counterstaining to show nuclei. Scale bar = 10 μm. The quantification graphs on the right show the average fluorescence intensity of each gene. Means ± SD, *p < 0.05, **p < 0.01 and ***p < 0.001.
