# Supplementary material for: Aged hippocampal single‐cell atlas screening unveils disrupted neuroglial system in postoperative cognitive impairment
Source: Aging Cell. 2024 Nov 14;24(3):e14406. doi: 10.1111/acel.14406 (PMC11896209; doi:10.1111/acel.14406)
Supplement: Supplementary file 2 — Tables S1–S2 [file ACEL-24-e14406-s001.docx]

**Table S1 The data quality control results**

| SampleName | CleanN | CleanGC | CleanQ20 |
| --- | --- | --- | --- |
| C1_S21_L003 | 0.00;0.00 | 46.56;45.51 | 98.95;96.79 |
| C1_S22_L003 | 0.00;0.00 | 46.53;45.51 | 98.90;96.36 |
| C1_S23_L003 | 0.00;0.00 | 46.56;45.55 | 98.91;96.59 |
| C1_S24_L003 | 0.00;0.00 | 46.55;45.50 | 99.00;96.61 |
| C1_S34_L001 | 0.00;0.00 | 46.47;45.37 | 98.41;95.67 |
| C1_S35_L001 | 0.00;0.00 | 46.52;45.44 | 98.46;95.97 |
| C1_S36_L001 | 0.00;0.00 | 46.52;45.39 | 98.56;95.95 |
| C2_S25_L003 | 0.00;0.00 | 46.67;44.77 | 98.97;96.93 |
| C2_S26_L003 | 0.00;0.00 | 46.68;44.83 | 98.91;96.74 |
| C2_S27_L003 | 0.00;0.00 | 46.67;44.81 | 98.88;96.67 |
| C2_S28_L003 | 0.00;0.00 | 46.64;44.78 | 98.90;96.35 |
| C2_S37_L001 | 0.00;0.00 | 46.63;44.66 | 98.50;96.32 |
| C2_S38_L001 | 0.00;0.00 | 46.65;44.76 | 98.47;96.21 |
| C2_S39_L001 | 0.00;0.00 | 46.63;44.70 | 98.41;96.04 |
| C2_S40_L001 | 0.00;0.00 | 46.48;44.57 | 98.26;95.46 |
| C3_S29_L003 | 0.00;0.00 | 46.45;45.73 | 98.92;96.10 |
| C3_S30_L003 | 0.00;0.00 | 46.45;45.63 | 98.96;96.70 |
| C3_S31_L003 | 0.00;0.00 | 46.44;45.60 | 98.92;96.90 |
| C3_S32_L003 | 0.00;0.00 | 46.45;45.66 | 98.90;96.60 |
| C3_S41_L001 | 0.00;0.00 | 46.42;45.61 | 98.46;95.48 |
| C3_S42_L001 | 0.00;0.00 | 46.40;45.50 | 98.48;96.00 |
| C3_S43_L001 | 0.00;0.00 | 46.41;45.51 | 98.45;96.24 |
| C3_S44_L001 | 0.00;0.00 | 46.41;45.57 | 98.43;95.97 |
| S1_S33_L003 | 0.00;0.00 | 46.76;44.53 | 98.93;96.77 |
| S1_S34_L003 | 0.00;0.00 | 46.78;44.58 | 98.93;96.89 |
| S1_S35_L003 | 0.00;0.00 | 46.76;44.62 | 98.90;96.36 |
| S1_S36_L003 | 0.00;0.00 | 46.77;44.58 | 98.94;96.75 |
| S1_S45_L001 | 0.00;0.00 | 46.68;44.41 | 98.42;96.08 |
| S1_S46_L001 | 0.00;0.00 | 46.74;44.50 | 98.46;96.24 |
| S1_S47_L001 | 0.00;0.00 | 46.69;44.50 | 98.39;95.68 |
| S1_S48_L001 | 0.00;0.00 | 46.74;44.47 | 98.50;96.15 |
| S2_S33_L004 | 0.00;0.00 | 46.46;45.03 | 98.82;96.37 |
| S2_S34_L004 | 0.00;0.00 | 46.46;44.99 | 98.84;96.82 |
| S2_S35_L004 | 0.00;0.00 | 46.48;44.97 | 98.84;97.01 |
| S2_S36_L004 | 0.00;0.00 | 46.46;45.00 | 98.82;96.81 |
| S2_S53_L001 | 0.00;0.00 | 46.47;44.98 | 98.41;95.68 |
| S2_S54_L001 | 0.00;0.00 | 46.10;44.73 | 98.08;95.65 |
| S2_S55_L001 | 0.00;0.00 | 46.44;44.93 | 98.46;96.33 |
| S2_S56_L001 | 0.00;0.00 | 46.45;44.96 | 98.48;96.19 |
| S3_S13_L001 | 0.00;0.00 | 46.94;46.57 | 98.99;97.01 |
| S3_S14_L001 | 0.00;0.00 | 46.93;46.51 | 98.99;97.24 |
| S3_S15_L001 | 0.00;0.00 | 46.94;46.60 | 98.97;97.03 |
| S3_S16_L001 | 0.00;0.00 | 46.93;46.58 | 98.96;97.03 |
| S3_S1_L003 | 0.00;0.00 | 46.93;46.60 | 98.94;96.59 |
| S3_S2_L003 | 0.00;0.00 | 46.93;46.53 | 98.95;96.85 |
| S3_S3_L003 | 0.00;0.00 | 46.93;46.63 | 98.91;96.61 |
| S3_S4_L003 | 0.00;0.00 | 46.92;46.60 | 98.92;96.60 |

CleanN: The number of undetermined bases; CleanGC: GC content; CleanQ20: quality score of bases greater than 20.

**Table S2 The top DEGs of major functional clusters in Figure 2-4**

|  | DEGs | avg_logFC | p value | Proportion of involved terms |
| --- | --- | --- | --- | --- |
| Metabolic Alteration | | | | |
| Neuron | *Ttr* | -2.1076095 | 3.85E-75 | 9.7% |
|  | *Atf3* | 0.52697159 | 6.72E-06 | 38.7% |
|  | *Mdk* | 0.50120684 | 0.0008524 | 38.7% |
|  | *Cdk2ap1* | 0.4274941 | 0.0048864 | 32.3% |
|  | *Ppib* | 0.41425855 | 0.0003197 | 9.7% |
| Astrocytes | *Dio2* | -0.3618534 | 3.66E-11 | 7.5% |
|  | *Neat1* | 0.33686843 | 9.24E-06 | 12.5% |
|  | *Msmo1* | -0.3032803 | 1.66E-09 | 5.0% |
|  | *Actg1* | 0.27863132 | 6.35E-11 | 7.5% |
|  | *Tcf25* | 0.26726379 | 0.00006 | 10.0% |
| Oligodendrocytes | *Apod* | 0.62148158 | 2.7E-58 | 8.1% |
|  | *Psap* | 0.40730377 | 1.13E-84 | 13.5% |
|  | *Rps24* | -0.251497 | 2.8E-89 | 10.8% |
|  | *Jund* | 0.2297389 | 1.12E-75 | 5.4% |
|  | *Gstm1* | 0.22476385 | 1.99E-22 | 8.1% |
| Neurotoxicity | | | | |
| Neuron | *Jun* | 0.82203573 | 2.957E-09 | 33.3% |
|  | *Neurod1* | 0.80153722 | 2.124E-05 | 33.3% |
|  | *Egr1* | 0.74080467 | 4.213E-07 | 25.0% |
|  | *Gfap* | 0.57262601 | 3.954E-05 | 8.3% |
|  | *Sox11* | 0.41052207 | 0.0244892 | 8.3% |
| Neurogenesis and Gliogenesis | | | | |
| Neuron | *Fos* | 0.80045903 | 2.296E-05 | 7.1% |
|  | *Ier2* | 0.70499282 | 2.302E-08 | 28.6% |
|  | *Nr2f1* | 0.48318596 | 0.00017 | 28.6% |
|  | *Ncdn* | -0.4701753 | 0.0291288 | 28.6% |
|  | *Nfia* | 0.46668379 | 0.0077664 | 7.1% |
| Inflammation | | | | |
| Microglia | *Ptgds* | 0.65079211 | 9.42E-180 | 6.0% |
|  | *S100a9* | 0.58427985 | 5.073E-23 | 12.0% |
|  | *Ifi30* | 0.32053112 | 2.147E-59 | 2.7% |
|  | *Lst1* | -0.320106 | 6.959E-66 | 6.0% |
|  | *Irf2bp2* | -0.290042 | 7.743E-61 | 0.7% |
| Stress and Apoptosis | | | | |
| Microglia | *Erdr1* | 0.36854899 | 8.694E-92 | 2.0% |
|  | *Apod* | 0.34598346 | 2.673E-87 | 2.0% |
|  | *Picalm* | -0.2958184 | 7.132E-79 | 6.1% |
|  | *Crybb1* | -0.2932425 | 1.914E-44 | 2.0% |
|  | *Irf2bp2* | -0.290042 | 7.743E-61 | 2.0% |
| Astrocytes | *Egr1* | 0.463642 | 8.62E-18 | 62.5% |
|  | *C1qa* | 0.46109449 | 5.96E-09 | 37.5% |
|  | *Fos* | 0.36064183 | 1.52E-08 | 37.5% |
|  | *Cx3cr1* | 0.29076424 | 5.52E-11 | 87.5% |
|  | *Mag* | 0.28188635 | 1.69E-07 | 68.8% |
| Rhythm Regulation | | | | |
| Microglia | *Dbp* | 0.36512923 | 1.74E-143 | 25.0% |
|  | *Fosb* | -0.35355004 | 7.45E-49 | 8.3% |
|  | *Apoe* | -0.31196197 | 0.045092 | 8.3% |
|  | *Cebpb* | -0.30735629 | 3.22E-66 | 8.3% |
|  | *Nr4a1* | -0.30285712 | 2.57E-38 | 8.3% |
| Astrocytes | *Dbp* | 0.68208328 | 3.4E-38 | 9.1% |
|  | *Egr1* | 0.463642 | 8.62E-18 | 36.4% |
|  | *Hexb* | 0.40591094 | 7.45E-07 | 9.1% |
|  | *Jund* | 0.39974373 | 1.52E-09 | 9.1% |
|  | *Id4* | -0.3955872 | 1.52E-16 | 9.1% |
| PAPs Plasticity | | | | |
| Astrocytes | *Slc1a3* | -0.3072342 | 1.86E-11 | 21.7% |
|  | *Cx3cr1* | 0.29076424 | 5.52E-11 | 65.2% |
|  | *Nnat* | -0.2861796 | 1.71E-06 | 13.0% |
|  | *Selplg* | 0.28570972 | 1.88E-09 | 21.7% |
|  | *Kif5a* | 0.26681383 | 4.53E-08 | 8.7% |
| Axonal Myelination | | | | |
| Oligodendrocytes | *Sgk1* | -0.5248613 | 4.76E-177 | 16.7% |
|  | *Creb5* | 0.27576083 | 3.79E-47 | 10.0% |
|  | *Uba52* | -0.2262999 | 3.02E-42 | 10.0% |
|  | *Rpl35* | -0.2213982 | 1.75E-44 | 10.0% |
|  | *Cacybp* | -0.2167667 | 1.1E-33 | 10.0% |
